# From Wide- to Low-Bandgap Semiconductors for Transient Photocurrent THz Emission: A Review

**DOI:** 10.3390/ma19143153

**Published:** 2026-07-22

**Authors:** Sanjit Varma, Tsuneyuki Ozaki, My Ali El Khakani

**Affiliations:** Institut National de la Recherche Scientifique, Centre Énergie Matériaux Télécommunications, 1650, Boulevard Lionel-Boulet, Varennes, QC J3X 1P7, Canada

**Keywords:** low-bandgap materials, wide-bandgap materials, semiconductors, germanium, transient photocurrent, THz sources, ultrafast photonics

## Abstract

Terahertz (THz) radiation generated through ultrafast transient photocurrent mechanisms has become a cornerstone of modern THz photonics, enabling broadband coherent emission with sub-picosecond temporal resolution. This review provides a comprehensive and mechanism-driven analysis of THz pulse generation via photo-Dember diffusion currents, surface depletion field acceleration, and biased photoconductive antenna architectures. We present a comprehensive comparative analysis of wide- and low-bandgap material platforms, including III–V, II–VI, and group IV semiconductors, as well as two-dimensional materials, topological insulators, and Weyl semimetals, highlighting how their intrinsic properties, such as band structure, carrier mobility, recombination dynamics, doping, and dielectric response, govern their THz emission efficiency, bandwidth, and spectral tunability. Special emphasis is placed on germanium (Ge), which has re-emerged as a highly promising THz source material owing to its high carrier mobility, long diffusion lengths, strain-tunable band structure, and CMOS compatibility. We highlight the roles of doping, strain-induced direct transitions, and several fabrication techniques in controlling the nonlinear photoexcited charge-carrier dynamics in Ge, thereby unlocking enhanced broadband THz performance. Finally, we explore the emerging application prospects of THz radiation, ranging from non-invasive security screening to biochemical sensing and archeological preservation. By bridging fundamental material science with scalable device architectures, this review outlines current challenges, highlights evolving opportunities in novel materials, and charts future directions towards integrated THz technologies.

## 1. Introduction

**Figure 1:**
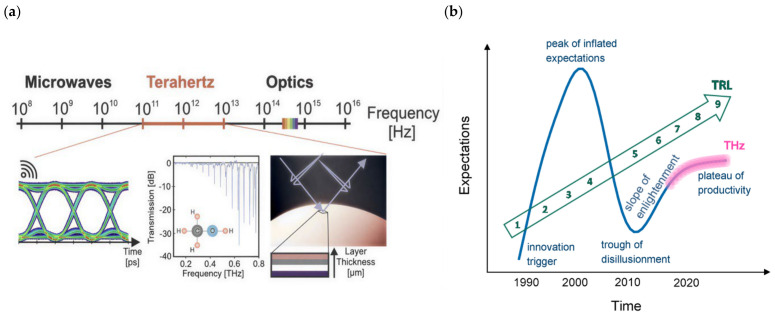
(**a**) The electromagnetic spectrum with corresponding key applications of THz, such as wireless communication (**left**), spectroscopic fingerprinting (**middle**), and non-destructive testing (**right**) [1]. (**b**) The Gartner Hype Cycle and the current envisaged position of THz technologies [3]. Reproduced from Refs. [1,3] under CC BY 4.0.

The terahertz (THz) spectral domain, loosely defined as the frequency range of 0.1–10 THz, bridges the technological gap between microwave and optics, as shown in Figure 1a [1]. Historically known as the “THz gap” due to early challenges with coherent radiation, this gap has now been largely bridged by advances in THz photonics over the last decade [2]. An illustration of this technological progress can be inferred from the Gartner Hype Cycle [3], shown in Figure 1b, which places several THz-based technologies in the “plateau of productivity,” indicating growing technological adoption and commercial interest. It is important to note, however, that the technology readiness level (TRL) varies across different THz applications, and that the Gartner Hype Cycle is not a rigorous measure of scientific maturity. Rather, it suggests that several THz-based systems (with TRLs of up to 8–9) are transitioning toward broader practical implementation and industrial relevance [3]. The primary impetus for the swift advancement in THz research is the ability of THz radiation to resonate with various fundamental motions of ions, electrons, and electron spins across all phases of matter [4]. Photon energies in the millielectronvolt (meV) range, corresponding to THz frequencies, represent an optimal frequency regime for coherent monitoring of electromagnetic (EM) fields without the need for an interferometer, thereby enabling high-resolution studies in both the temporal and spectral domains. This dual capacity enables a direct observation of the ultrafast temporal evolution of optical response functions on the femtosecond (fs) to picosecond (ps) timescales, offering profound insight into the carrier dynamics of bound and free charge carriers, confined plasmas [5], excitonic states [6], and transient molecular dipoles [7]. Moreover, the noncontact nature of THz spectroscopy is well suited to extreme experimental conditions, including high magnetic fields [8], cryogenic temperatures [9], and intense electric fields [10]. It is also very convenient for non-contact probing methods, such as polarization-resolved techniques, which are essential for materials where electrical contacts are either impractical or undesirable [11].

Moreover, in semiconductor nanostructures, the THz frequencies correspond to energy-level splitting due to quantum confinement [12] and magnetic-field-induced separations [13], enabling the exploration of orbital [14] and spin-state dynamics [15], characterization of interfaces [16], subsurface defects [17], coherent quantum control [18], and superposition states of carriers [19]. Being exceptionally responsive to low-energy excitations [16], THz radiation tends to dictate the low-energy vibrational phonon modes in organic and inorganic crystals [20], intermolecular vibrations in weakly bonded molecular solids [21], and relaxational dynamics in aqueous [22] and hydrated biological environments [23]. The THz emission has significantly advanced in recent decades, evolving from rather bulky, less efficient systems to highly tunable and compact sources [24]. Initial THz emitter sources were largely constrained by technological limitations, primarily relying on electrical or optical down-conversion methods. A few solid-state devices, such as IMPATT (Impact Ionization Avalanche Transit-Time) [25], resonant tunneling diodes (RTDs) [26], and Gunn diodes [27], were among the earliest continuous-wave (CW) THz emitters operating in the upper microwave and lower THz regions. Nonetheless, on the lower-frequency side (up to 100 GHz), their output power was constrained by the transit time of electrons and thermal losses [28].

Nonetheless, several nonlinear optical crystals, including both inorganic (e.g., LiNbO_3_ [29], ZnTe [30], GaP [31], GaAs [32]) and organic (e.g., DAST, DSTMS, OH1, HMQ-TMS, and BNA) materials [33] have been instrumental in advancing the THz field. Upon excitation with an ultrafast pulsed laser, these materials generate broadband THz radiation either by optical rectification (OR) [34] or by difference frequency generation (DFG) [20]. Their ability to convert high-intensity near-IR or visible light pulses into THz frequencies using second-order nonlinear optical processes rendered them essential in early time-domain spectroscopy. While nonlinear optical crystals provided an early platform for coherent THz generation, their reliance on stringent phase-matching [35], bulk geometries [36], three-photon absorption (3PA) of the near-infrared radiation, and material dispersion/absorption properties [37] poses certain limitations in terms of their efficiency [38], tunability [39], and device integration [40].

In parallel, recent years have seen significant advances in the understanding and implementation of semiconductors in modern-day THz research, instigating a paradigm shift from the typical THz dynamics driven by nonlinear optical crystals. Broadband THz radiation using low- and wide-bandgap materials has always been a complex, multi-parameter phenomenon primarily influenced by the intrinsic material properties such as band structure [41], photo-excited charge-carrier mobilities [42], doping concentrations [43], electrical conductivity, carrier recombination rate [44], defect densities [45], and crystallinity [46]. An early breakthrough occurred in 1984 [47], when free-space THz pulses were demonstrated using time-varying Hertzian dipoles excited by fs lasers, thereby enabling broadband THz pulse generation and detection via ultrafast carrier acceleration. Optical excitation of semiconductors such as GaAs [46], InAs [48], InP [49], and more recently Si [50] and Ge [51,52] using ultrafast fs-pulsed laser sources leads to broadband coherent THz radiation via different mechanisms [53]. These emission mechanisms broadly include transient photocurrent generation via diffusion current-induced photo-Dember (PD) effect [54], drift current-induced surface depletion, field-induced acceleration [55], and electric field-induced optical rectification (EFIOR) attributed to higher-order nonlinearities [56]. The incorporation of these materials into PCAs and Auston switches has significantly enhanced the feasibility of THz systems, promoting their extensive application in time-domain spectroscopy, near-field imaging, and on-chip diagnostics [41,57]. In addition to bulk semiconductors, recent advancements in thin-film deposition techniques with their nanostructured surfaces, nanowires, metamaterials, heterostructures, and low-dimensional 2D materials have further widened the scope of THz radiation [16,44,58]. Quantum wells [59], superlattices [60], and cascaded heterostructures, particularly using III–V materials such as GaAs/AlGaAs, can also be used as tunable, narrowband THz sources [61]. On the other hand, 2D materials such as graphene, with their massless Dirac fermions and ultrafast carrier relaxation times, exhibit broadband THz emission through hot-carrier dynamics corresponding to intraband free-carrier absorption [62]. Other 2D materials tailored for THz radiation include transition metal dichalcogenides (TMDCs), black phosphorus (BP), Weyl semimetals (WSMs), and organic-inorganic perovskites [63,64]. These materials have garnered significant attention due to their unique electronic, optical, and mechanical properties [16]. Although these materials have generated promising future opportunities for tunable THz emission, their effective integration into large-scale, robust device platforms still remains a technical challenge, primarily due to material stability and fabrication complexity issues.

Furthermore, group IV semiconductors, particularly Si, Ge, and their alloys [65], offer a compelling balance between performance, scalability, and technological maturity. Recent advancements in material deposition technologies, strain engineering, and controlled doping strategies have further renewed interest in these materials. Although Si-based devices, with their excellent CMOS compatibility, have long dominated the microelectronics industry, an ever-growing demand for integrated photonic devices for other ultrahigh-speed optoelectronic applications has significantly increased the appeal for Ge-based THz radiation sources. A few reports have even suggested that heavily doped [54], ion-irradiated [66], and noble-metal ion-implanted Ge [67] can sufficiently provide trap-induced defects to further eliminate any slow charge carriers that are generated during the phonon-assisted recombination process. This would ensure complete recombination of electron–hole pairs within the duration of the fs laser pulse.

Nonetheless, Ge has proven to be a prominent material of choice for advanced integrated photonics, especially for near- and mid-infrared (IR) photodetectors [68]. Apart from THz emission via surface-field depletion and photo-Dember effects, recent studies have also shown third-order nonlinear susceptibility (χ^(3)^) response from Ge in the mid-IR region, which is even four times higher than that of Si [55]. Besides their application in ultrafast nonlinear THz research, the distinctive optoelectronic characteristics of Ge make it a candidate of choice for active modulation and switching in high-speed signal processing, effectively bridging the gap between electronic and photonic applications. Unlike other wide-bandgap materials, Ge can efficiently generate photoexcited charge carriers using readily available laser sources operating at central wavelengths of 1045 or 1550 nm, making it well-suited for telecom-compatible THz systems [67]. Moreover, compatibility with CMOS technologies facilitates seamless integration of Ge into Si-based platforms, thereby opening opportunities for practical, on-chip THz device implementation. Other innovative device architectures, such as vertical Si-graphene-Ge transistors, have demonstrated Ge’s THz operational capabilities with enhanced on-current and reduced parasitic capacitances, rendering them suitable for high-speed logic and RF front ends [69]. Similarly, Ge-based field-effect transistors (FETs), including Ge-core/a-Si-shell nanowire FETs, have also shown excellent responsivity and low noise-equivalent power in the THz regime, positioning them as viable contenders for ultra-sensitive detectors and high-frequency analog circuits [70]. Altogether, the effective incorporation of low-bandgap materials as ultrafast THz devices fundamentally relies on the ability to fabricate high-quality materials with precise control over their structure, morphology, strain, crystallinity, electrical conductivity, and doping level.

It is worth recalling that several review articles have recently covered different aspects of THz science and technology. For example, Burford et al. [71] and Castro-Camus et al. [72] focused primarily on photoconductive antennas, photoconductive switches, and related device architectures. Other reviews discussed THz generation using emerging quantum materials, symmetry-broken systems, topological insulators, van der Waals materials, graphene, MXenes, TMDCs, and metamaterials [41,73,74]. In addition, the 2023 THz Science and Technology Roadmap has provided a broad overview of advances in THz sources, detectors, imaging, communications, and applications [75]. Nevertheless, these papers do not provide a unified mechanism-driven comparison of THz emission across these material classes. The present review aims to fill this literature gap with a twofold objective: (i) to provide a unified, mechanism-driven comparative framework for THz emission across a broad range of semiconducting materials, enabling a direct discussion of the underlying physical processes governing THz generation rather than focusing on a specific device architecture or material class; and (ii) to offer a dedicated and comprehensive assessment of group IV materials, particularly Ge-based THz emitters, owing to their increasing relevance in integrated THz photonics [76]. We begin by exploring the fundamental transient photoconductive mechanisms underlying the emission of single-cycle THz pulses, with particular emphasis on the roles of surface- and bulk-related phenomena. By comparing different key materials and their properties of interest, we highlight their respective advantages and limitations for numerous THz applications. The materials considered include those from groups III–V, II–VI, and IV, as well as two-dimensional (2D) and layered materials, topological insulators (TIs), and Weyl semimetals (WSMs). Particular emphasis is placed on group IV materials, especially Ge, for which we examine its unique emission characteristics, nonlinear charge-carrier dynamics, recent progress in its growth by advanced deposition techniques, and its implementation into functional THz-based devices. Finally, we discuss the broader technological landscape and some potential applications of THz in areas ranging from non-destructive testing and biochemical sensing to art and archeological preservation.

## 2. Transient Photocurrent-Based THz Emission

In general, the photoconductive broadband THz emission in semiconductors is dependent on the physical mechanisms arising from the ultrafast dynamics of photoexcited charge carriers, along with their ultrashort interactions with internal or external fields when excited by a pulsed fs laser source [77]. The temporal evolution of the radiated THz electric field component $E_{THz}\left(t\right)$ is proportional to the second temporal derivative of the dipole moment $\vec{p}\left(t\right)$, or the first temporal derivative of the transient current density $J\left(t\right)$, evolving over a period of several hundred fs to a few ps [78]. This is the fundamental principle behind the generation of broadband EM transients with frequencies in the THz range using ultrashort fs laser pulses, and is generally defined as follows:

(1) $$E_{THz}\left(t\right)\propto \frac{d^{2}\vec{p}\left(t\right)}{{dt}^{2}}\propto \frac{dJ\left(t\right)}{dt}$$

The frequency components of this EM field are thus associated with the dynamics of the transient current that radiates broadband THz electric fields. Unlike conventional radiation sources, semiconductor-based THz emitters can exploit a rich interplay of drift, diffusion, and nonlinear optical processes to convert fs optical pulses or applied bias fields into broadband THz radiation. Different emission mechanisms can thus be broadly classified into three categories: (i) biased PCA emission [79]; (ii) carrier diffusion effects such as the photo-Dember effect [80]; and (iii) drift-carrier current-induced surface field-depletion emission [44]. The significance of these generation mechanisms essentially relies on the choice of substrate material, excitation conditions, and device architecture. Concise explanations of the different underlying physical processes responsible for THz emission in semiconductors are presented in the following subsections.

### 2.1. Photo-Dember Effect

**Figure 2:**
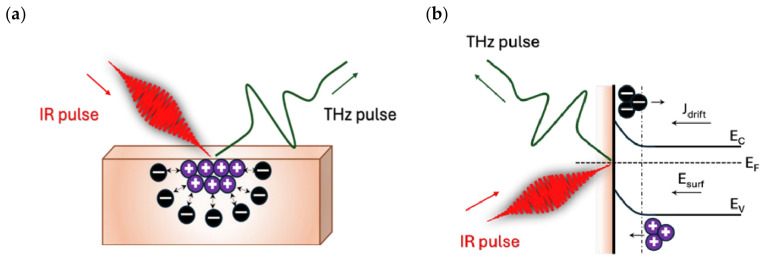
Schematic illustration of THz emission using the (**a**) photo-Dember (PD) effect and (**b**) surface depletion field.

The photo-Dember (PD) effect is an ultrafast quasi-ballistic high-energy-carrier-transport phenomenon responsible for THz emission in semiconductors, especially with high carrier mobility and significant differences between electron and hole mobilities [81,82,83]. Upon excitation with an ultrafast fs laser pulse, a semiconductor surface imposes a boundary condition on the diffusion current, as carriers cannot go beyond the semiconductor-air interface. The boundary condition constrains the photoexcited charge carriers and provides a net direction (into the bulk) to generate a localized transient electric dipole moment due to a non-equilibrium distribution of electrons and holes, thus radiating THz electric fields [84], as illustrated in Figure 2a. In general, the radiated THz emission via the PD effect propagates perpendicularly and with cylindrical symmetry to the optical pump [84]. The THz radiation by surge diffusion current, associated with the hot photocarrier injection with the excitation energies far above the conduction bands, is often expressed as [85]:

(2) $$E_{THz}\propto \frac{\partial J\left(t\right)}{\partial t}\propto I_{p}\sqrt{\frac{{(E}_{p}-E_{g})}{m^{*}}}$$

Here, $I_{p}$ is the laser intensity, ($E_{p}-E_{g}$) signifies the acquired velocity from the excess photon energies, and $m^{*}$ is the effective mass. The PD effect is highly efficient for narrow-bandgap III–V semiconductors, such as InAs and InSb, where the low effective mass and high electron mobility lead to the ultrafast buildup and relaxation of the photo-Dember field [86]. In addition, the radiated THz emission exhibits strong anisotropic properties that are broadly dependent on random scattering events, the effective mass, band structure, lattice vibrations, and the carrier–carrier scattering time in the material. Additionally, the lateral photo-Dember effect has also been reported in GaAs-based THz emitters using the drift-diffusion equations [84,87].

### 2.2. Surface Depletion Field-Induced THz Emission

Another significant mechanism contributing to photocurrent-induced THz radiation is the surface depletion field-induced THz emission, arising from the intrinsic built-in or external electric field near the surface of the semiconductor [81]. The THz electric fields, as illustrated in Figure 2b, originate from carrier acceleration due to band bending in either the surface depletion or accumulation layer generated due to Fermi-level pinning or surface states that trap charges and modify the local electrostatic potential [81,84]. The optical excitation is typically incident at an angle with respect to the semiconductor surface to ensure effective collection of the THz radiation. The internal built-in electric field, directed normal to the surface, rapidly accelerates the electron–hole pairs (in opposite directions) that are generated at the surface of the semiconductor upon excitation by a fs laser pulse [88]. These accelerated charge carriers generate a transient surge drift current, $J\left(t\right)$, which depends on surface potential, depletion layer thickness (w), diffusion voltage ($V_{D}$), mobility (μ), and optical penetration depth ($\lambda_{L}$) of the material. The time derivative of this surge photocurrent is the source of the THz radiation, which is often described as [85]:

(3) $$E_{THz}\propto \frac{\partial J\left(t\right)}{\partial t}\propto \frac{∆n∆v}{∆t}\propto \mu E_{B}I_{p}$$

Here, $E_{B}$ is the built-in electric field. For w > $\lambda_{L}$, Equation (3) can be written as:

(4) $$E_{THz}\propto \pm \;\mu \sqrt{\frac{N_{i}V_{D}}{\varepsilon_{r}}}I_{p}$$

where $N_{i}$ and $\varepsilon_{r}$ are the impurity density and dielectric constant of the semiconductor, and $I_{p}$ is the laser intensity. For most of the doped semiconductors, with w ≤ $\lambda_{L}$, Equation (4) is further simplified as:

(5) $$E_{THz}\propto \pm \;\mu \frac{V_{D}}{\lambda_{L}}I_{p}$$

The polarity of the emitted THz radiation can be tuned by engineering the type of doping (n-type or p-type) [81] or according to the band-bending direction near the surface for the non-doped/semi-insulating semiconductors [85]. Moreover, materials such as GaAs, InP, and Si can exhibit prominent surface depletion fields due to native oxide layers or surface traps, especially during low-temperature growth or specific doping profiles. Other materials, such as low-temperature-grown GaAs (LT-GaAs) with ultrafast carrier trapping times (sub-ps) [89] show higher optical-to-THz conversion efficiencies and significantly larger breakdown fields, thereby enhancing the temporal resolution and bandwidth of the emitted THz radiation [90]. In the case of Ge, reports suggest that the THz emission mechanisms are an interplay of the photocurrent surge in the surface electric field and the PD effect [55]. However, the co-existence of drift and diffusion currents near the surface makes it difficult to study the mechanisms separately [85]. This ambiguity is particularly evident in Ge-based THz emission, as it originates from the same ultrafast photoexcited transient carriers but reflects the competition between diffusion-driven carrier separation (photo-Dember effect) and drift acceleration in surface depletion fields. The relative weight of these contributions is expected to vary depending on material parameters (such as degree of crystallinity, doping level, surface condition, and carrier mobility) as well as on excitation conditions (including wavelength and absorption depth). Consequently, the same physical system can exhibit different dominant emission behaviors under different experimental configurations. As a result, no universal consensus has yet emerged regarding the dominant THz emission mechanism in Ge-based structures. Further systematic studies involving controlled variations in different Ge architectures are still essential to reach a definitive understanding of the dominant mechanism.

### 2.3. Biased Photoconductive Antenna (PCA)-Based THz Emission

**Figure 3:**
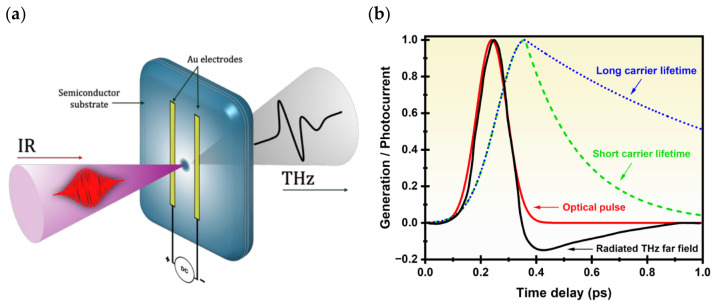
(**a**) Schematic illustration of THz emission using a biased dipole PCA. (**b**) Typical temporal profile of an ultrafast fs optical pulse (red trace) and the generated photocurrent in the antenna gap for photoconductive material with either short-carrier lifetimes (green trace) or long-carrier lifetimes (blue trace), leading to a far-field single-cycled THz pulse (black trace).

The biased PCA is a widely used method for generating broadband THz radiation using semiconductors under the influence of a static bias field. Upon excitation by an fs laser pulse, the PCA instantaneously excites the electron–hole pairs within the small photoconductive gap of the antenna, as shown in Figure 3a. These photo-excited charge-carriers are further accelerated in opposite directions, towards the metallic electrodes, by the applied external DC electric field. The efficiency of a biased PCA is often dictated by the characteristics of the photoconductive substrate, such as its carrier lifetime, band gap, breakdown voltage, resistivity, and electron mobility [71,88,91]. The rapid rise in photoinduced time-dependent charge-carrier density, followed by a subsequent decay governed by charge recombination and trapping, results in a typical single-cycle pulsed THz radiation with a broad spectrum, as illustrated in Figure 3b. Materials such as GaAs [92], ZnSe [93,94], radiation-damaged Si on sapphire (RD-SOS) [95], nanocrystalline Si [96], and organic semiconductors such as highly oriented poly(p-phenylene vinylene) (PPV) [97] are among the most commonly used PCA materials for broadband THz emission due to their short carrier lifetime and high breakdown voltage.

**Figure 4:**
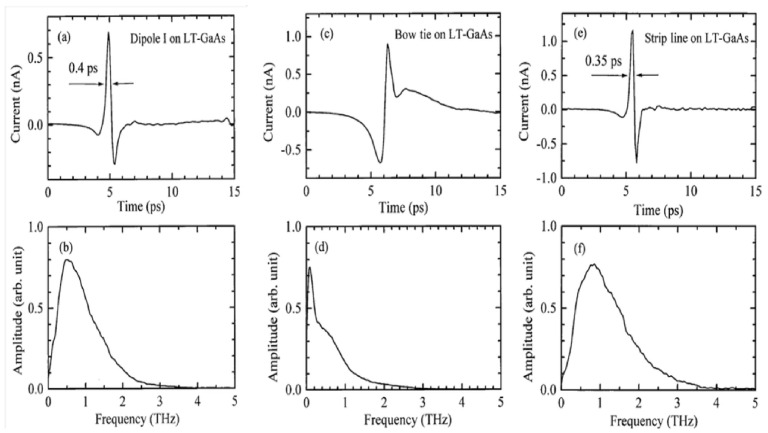
Radiated THz waveform and Fourier-transformed amplitude spectrum from LT-GaAs PCAs with (**a**,**b**) 30-μm dipole, (**c**,**d**) bowtie, and (**e**,**f**) strip-line geometries. Reproduced from Ref. [100] © Optica Publishing Group.

In addition, the antenna electrode configurations, such as dipole, bow-tie, log-periodic, or strip line structures, impact the impedance matching and emission bandwidth, with smaller inter-electrode gaps and sharp features enhancing the locally generated electric field [98,99,100,101]. For example, Figure 4 shows a comparison of the emitted THz waveform and its Fourier-transformed amplitude from the low-temperature (250 °C) epitaxially grown and post-annealed (at 600 °C) LT-GaAs-based PCAs with different PCA geometries, such as a 30 µm dipole, bowtie, and strip line [100]. In addition, recent advancements in plasmonic contact electrode gratings [102], nanoantenna-enhanced PCAs [101], and dielectric metasurfaces [103] have also demonstrated multi-fold improvements in photocarrier collection, emitted THz power, and bandwidth, making them a key component for next-generation high-performance THz photonics.

Novel semiconductors including 2D materials (e.g., graphene, MoS_2_) [104,105], topological insulators (e.g., Bi_2_Se_3_, Bi_1−x_Se_x_) [106,107], and narrow bandgap III–V compounds (e.g., InSb, InAs) [48,108] are being actively explored for their exceptional carrier mobilities, ultrafast carrier dynamics, and tunable optoelectronic properties. These materials exhibit strong nonlinear light–matter interactions, thereby enabling hot carrier transport and plasmon-assisted acceleration. Such non-traditional materials are pivotal in enhancing PCA-based THz technology for real-world applications such as THz imaging, spectroscopy, and high-speed communications. An in-depth analysis of these new THz-emitting materials is provided in the subsequent sections.

## 3. THz Radiation Using Different Semiconductors

Throughout the decades, a broad class of different materials, including nonlinear optical crystals, conventional semiconductor thin films, 2D nanostructures, and novel hybrid materials, have been investigated for their THz emission characteristics. Few reports suggest that the intrinsic efficiency of THz generation via optical means using difference frequency generation within a nonlinear crystal is often limited by the Manley–Rowe relation to ~10^−3^, since it involves the conversion of infrared to THz frequencies [1,109]. Nonetheless, to mitigate these limitations and to further improve the THz radiation efficiencies, PCAs provide a compelling option, as THz radiation from these sources originates from an externally applied bias field [110]. These can be further classified into two categories, depending on the applied photoconductive techniques, based on either the high-speed PCAs integrated with wideband radiating antennas or the bare nonlinear optical material surfaces illuminated by pulsed fs laser sources. While the underlying physical mechanisms, such as nonlinear electric field-induced optical rectification (EFIOR) [56], cold-plasma oscillations [54], photo-Dember effects [58,111], and surface depletion field-induced processes [44] remain the same in both classes of photoconductive techniques, the efficiency, bandwidth, and their output intensity are highly material-dependent.

A few key material parameters that directly influence the THz radiation process from different materials are their carrier lifetime (τ), mobility (μ), bandgap (E_g_), dielectric function ε(ω), nonlinear susceptibilities (χ^(2)^, χ^(3)^), and lattice symmetry. Nonetheless, each class of materials (III–V, II–VI, IV, 2D, layered, topological insulators, and Weyl semimetals) exhibits its own set of unique material properties crucial for efficient THz radiation. In the upcoming sections, we provide a more comprehensive and comparative survey of the broad spectrum of materials that have been explored for THz radiation, thereby bridging the gap between material physics and applied THz photonics while providing additional details on the material-specific advantages, their underlying mechanisms, and their design considerations.

### 3.1. Group III–V Materials

In general, III–V materials such as GaAs, GaN, InP, InAs, InSb, and their alloys exhibit direct bandgaps and ultrafast carrier dynamics (sub-ps photocarrier lifetimes) due to their high electron mobilities and strong nonlinear coefficients, thus making them suitable for efficient THz emission. Table 1 shows a non-exhaustive list of various III–V materials used for THz generation with their respective band gaps along with their reported carrier mobilities (at room temperature), dominant THz mechanisms, and key advantages and limitations. The THz radiation generated from those semiconductors naturally depends on their optoelectronic properties and the used laser excitation wavelength.

**Figure 5:**
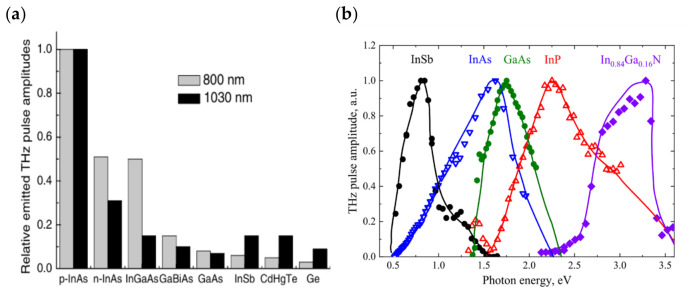
(**a**) Comparison of THz amplitudes emitted from different semiconductor surfaces after excitation by the fs optical pulses at two different wavelengths. (**b**) THz emission spectra of InSb, InAs, GaAs, InP, and In_0.84_Ga_0.16_N. Reproduced (**a**) and adapted (**b**) from Ref. [112] under CC BY 4.0.

For instance, Figure 5a compares the amplitudes of the THz pulses emitted from various semiconducting surfaces illuminated by either Ti:Sapphire (λ ~ 800 nm) or Yb-based (λ ~ 1030 nm) laser excitation sources. The difference in THz pulse amplitudes for different materials is due to their varying compatibilities with the two different excitation wavelengths [112]. This compatibility is reliant on the fact that materials with direct narrow band gaps and indirect wide band gaps have been reported to exhibit a superior THz emission efficiency at excitation wavelengths situated between the direct and indirect band gaps [113]. The dependence of emitted THz pulse amplitude on the excitation photon energy for different III–V materials is further analyzed via THz emission spectroscopy (TES), as shown in Figure 5b, where the maximum amplitude of the emitted THz pulse corresponds to the onset of electron transfer from Γ to L valleys in the conduction band. At relatively lower and higher photon energies (with respect to the maximum emission point), the THz generation is primarily due to the influence of the surface built-in electric field and the contribution of ballistic electron motion, respectively. Moreover, one can use the slopes of these curves to determine the energy (eV) distance from the conduction band minima to different valleys at the X and L points of the Brillouin zone [112].

**Table 1:** Comparison of different III–V materials used for THz radiation, including their band gaps, carrier mobilities (at room temperature), dominant THz emission mechanisms, and key advantages and limitations. In general, these reported values are process-dependent and may vary with growth conditions, doping or ion implantation, defects, and nanostructuring.

Material	Bandgap (eV)	Electron/Hole Mobility (cm^2^ V^−1^ s^−1^)	Dominant THz Mechanism(s)	Key Advantages	Key Limitations	Refs.
**GaAs**	~1.4	~7200–9000/ ~188–460	Photoconductive switching, surface-field emission	Mature PCA platform, high THz output	Expensive epitaxy, lifetime-mobility tradeoff	[114,115]
**GaN**	~3.4	~1030–1300/ ~18–50	Surface-field emission	High breakdown field, thermal stability	High-energy excitation, lower carrier mobility	[114,115]
**InP**	~1.3	~4000–5400/ ~170–650	Photoconductive switching, surface-field emission	Good photonic integration	Slower carrier dynamics than LT-GaAs	[86,116]
**InAs**	~0.3	~20,000–35,000/ ~240–530	Photo-Dember effect	Strong bias-free THz emission, high electron mobility	Low breakdown fields, toxic As gas handling	[86,116]
**InSb**	~0.17	~70,000–78,000/ ~600–850	Photo-Dember effect	Exceptional mobility, strong bias-free THz emission	Strong temperature dependence, narrow bandgap	[117,118]

**Figure 6:**
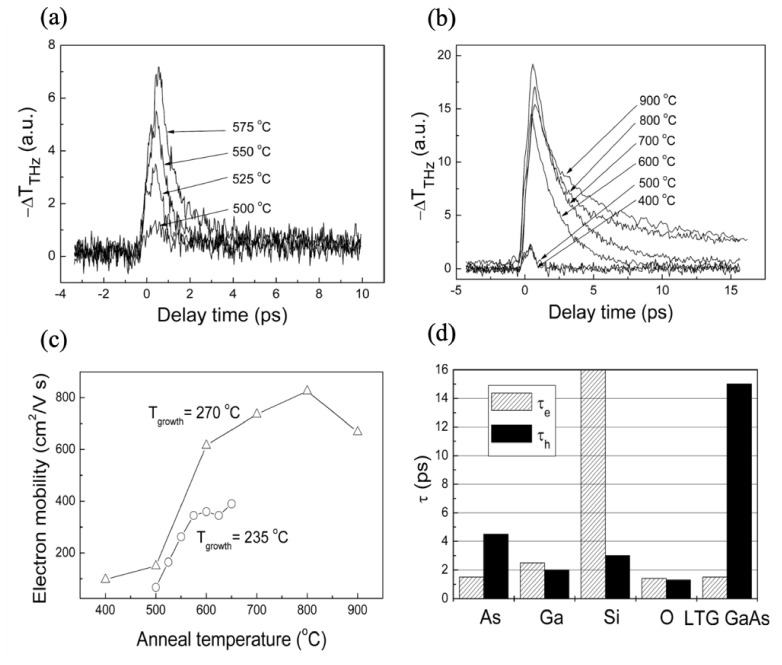
Time dependence of the THz differential transmission signal for the LT-GaAs sample grown at (**a**) 235 °C and (**b**) 270 °C and annealed at different temperatures. (**c**) Electron mobility of the LT-GaAs samples as a function of their deposition and annealing temperature. (**d**) Electron and hole trapping times of the LT-GaAs (grown at 270 °C) and GaAs implanted with As, Ge, Si, and O ions (implantation dose = 10^16^ cm^−2^). Reproduced from Ref. [121] under CC BY 4.0.

Gallium arsenide (GaAs) stands out as one of the most established materials for broadband THz radiation when excited at 800 nm using Ti:Sapphire fs lasers. Under ambient conditions, with a wide bandgap (~1.4 eV) and high electron mobility (~7000 cm^2^V^−1^ s^−1^) [119], GaAs is seen as the semiconducting material of choice for fabricating photoconductive antennas (PCAs), electro-optic (EO) sensors, and nonlinear optical devices. In particular, low-temperature-grown GaAs (LT-GaAs) is known for its ultrafast carrier recombination lifetimes (<10 ps) [120], which is crucial for generating short THz pulses with a broad spectrum. The recombination lifetime is generally dependent on the material elaboration process used (e.g., the deposition temperature of the epitaxial growth, the ex situ thermal annealing treatment, the ion implantation using different dopants, etc.). Figure 6a,b illustrate well how the shape of the THz differential transmission signal is directly affected by either the deposition (235 °C and 270 °C) or annealing (400 °C to 900 °C) temperatures in the case of epitaxially grown LT-GaAs [121]. Moreover, it has been observed that with longer carrier lifetimes, the THz pulse evolves from a nearly symmetric bipolar waveform to an asymmetrically bipolar or almost unipolar radiation. Such a unipolar waveform has been particularly reported in the case of semi-insulating GaAs, which is known for its long carrier recombination times (>100 ps) compared to LT-GaAs [122]. Consistently, the electron mobility in LT-GaAs layers has been shown to increase with processing conditions (growth and/or annealing temperature), reaching values up to ~800 cm^2^V^−1^ s^−1^, as shown in Figure 6c. For annealing temperatures higher than 800 °C, the observed drop in the electron mobility was attributed to the appearance of As precipitates, which act as additional electron scattering centers in LT-GaAs. On the other hand, the ion implantation of GaAs by different dopants (i.e., As, Ge, Si, and O, at a dose of 10^16^ cm^−2^) has been shown to modulate their electron and hole recombination dynamics (Figure 6d), thereby impacting their THz emission properties [121]. Nanostructuration of III–V semiconductors has also been reported as an interesting approach to influence their THz emission properties. Indeed, Al_0.2_Ga_0.8_As nanowires (~60 nm-diam.) grown via the vapor–liquid–solid (VLS) method have exhibited an increased THz emission efficiency because of better control over the bandgap and the photocarrier dynamics in these 1D materials [61].

Gallium nitride (GaN) is another interesting III–V semiconducting material, known for its very wide bandgap (~3.4 eV) and high breakdown field (~2 MV/cm), which has been investigated for the upper THz frequency band. Despite its lower electron mobility relative to GaAs or InAs, different GaN-based heterostructure devices have been reported as efficient THz emitter sources [115]. These devices include plasmonic GaN/AlGaN heterostructures [123], negative differential resistances (NDRs) [124], Schottky diodes [125], Impact Ionization Avalanche Transit-Time diodes (IMPATTs) [126], quantum cascade lasers (QCLs) [127], high electron mobility transistors [128], Gunn diodes, and tera field-effect transistors (FETs) [129].

In contrast, indium antimonide (InSb), with its extremely narrow bandgap (~0.17 eV), is particularly interesting for its capacity to generate nonlinear THz due to its remarkably high electron mobility (~78,000 cm^2^V^−1^ s^−1^) [118]. Moreover, at appropriate azimuthal angles, a significant temperature dependence of the THz radiation amplitude is also observed for InSb [130]. Along with ultrafast photoinduced current dynamics, InSb-based THz emitters have shown exciting results towards developing novel high-performance millimeter- and THz-wave optoelectronic devices generating strong THz transients significantly enhanced by the plasmonic mechanisms, including surface plasmon resonance [131], Smith–Purcell effects [132], and magnetoplasmon polaritons [133]. Analogous to InSb, indium arsenide (InAs) is another III–V material extensively used for THz emission due to its relatively narrow direct bandgap (~0.36 eV) and a relatively high electron mobility (~22,700 cm^2^V^−1^ s^−1^), compared to those of GaAs and GaN. Unlike GaAs, InAs generates THz radiation predominantly through the photo-Dember effect, where asymmetric carrier diffusion driven by large electron–hole mobility differences leads to a transient dipole perpendicular to the surface [86]. This mechanism enables efficient THz emission even without an applied bias, making InAs favorable for compact and bias-free THz sources. Finally, indium phosphide (InP), with its intermediate bandgap (~1.34 eV) and moderate carrier mobilities (see Table 1), is also used as a PCA substrate and for electro-optic (EO) sampling. However, compared with LT-GaAs, it generally exhibits slower carrier dynamics and stronger nonlinear behavior, which is often explained by a two-photon excitation model [134]. Moreover, its implementation in efficient PCAs requires further bandgap engineering to meet the ultrafast performance demands of broadband THz applications.

Despite their outstanding THz emission performance, III–V semiconductors are not without limitations. The fabrication of high-quality III–V materials with low defect densities often relies on sophisticated epitaxial growth techniques, resulting in increased manufacturing complexity and cost. Moreover, challenges associated with wafer-scale integration, long-term device stability, and compatibility with mature CMOS technologies continue to hinder their widespread deployment [135]. While narrow-bandgap materials such as InAs and InSb exhibit exceptionally high carrier mobilities and strong THz emission efficiencies, incorporating hazardous, toxic precursors such as As and Sb introduces environmental and handling concerns during large-scale device fabrication. Despite remaining the most widely used materials for THz radiation, these fabrication challenges have motivated a search for alternatives that offer a more favorable balance between THz efficiency, cost-effectiveness, and technological compatibility.

### 3.2. Group II–VI Materials

**Figure 7:**
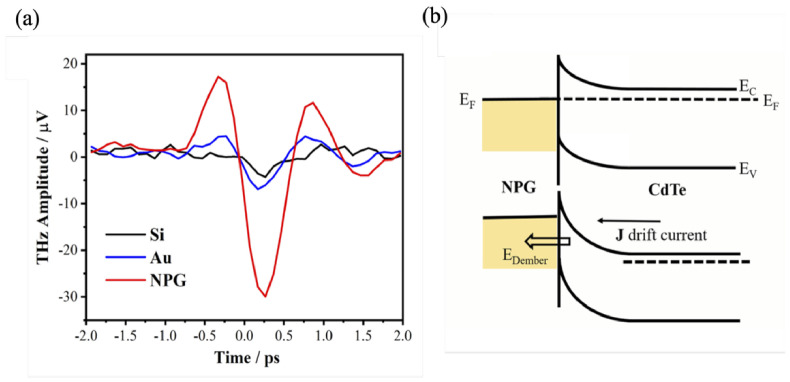
(**a**) Emitted THz spectrum of amorphous CdTe thin film over Si, Au, and nanoporous Au (NPG) substrates. (**b**) Schematic energy band diagram of the CdTe/NPG junction and the surface emission field after optical illumination with 780 nm. Reproduced from Ref. [139] © Optica Publishing Group.

Recently, II–VI materials have been increasingly exploited in THz photonics owing to their favorable nonlinear optical properties and strong electro-optic responses. These materials even possess high damage thresholds, low THz phonon absorption, and broad optical transparency in the near- to mid-IR frequencies. Even though zinc telluride (ZnTe), with a wide bandgap of ~2.26 eV, has been established as a standard electro-optic (EO) crystal [136], it has not been further explored for photoconductive-based coherent THz emission and detection due to the observed phase mismatch resulting from the birefringence and group velocity dispersion at sub-THz frequencies [137]. Nonetheless, other group II–VI materials such as cadmium telluride (CdTe), mercury telluride (HgTe), mercury cadmium telluride (MCT), and zinc selenide (ZnSe) have been investigated either as thin films, nanocrystals (NCs), or as large-aperture PCAs (LAPCAs) in order to generate and detect THz radiation via transient current surge methods efficiently. In fact, the use of a CdTe crystal as an efficient THz emitter, pumped via a Ti:Sapphire laser, is often limited by its distinct direct bandgap of ~1.48 eV, causing strong absorption at λ ~800 nm, thereby diminishing the effectiveness of the optical rectification considerably [138]. To overcome this limitation, CdTe has been used as amorphous thin films (less than 20 nm thick) thermally evaporated onto various substrates (including Si, Au, 3D-nanoporous gold (NPG) substrate, and AuAg alloy (Au_25_Ag_75_, wt%)) [139,140]. As shown in Figure 7a, THz emission from CdTe deposited over NPG is ~10 times and ~7 times stronger than that of CdTe deposited on Si and on flat Au, respectively [139]. Similarly, THz emission from CdTe over Au_25_Ag_75_ alloy substrates is fivefold stronger than its counterpart on Si substrates. The primary THz emission mechanism in CdTe films supported by plasmonic metals is attributed to the transient photocurrents induced either by the intrinsic band bending at the surface depletion field (drift currents) or the photo-Dember effect (diffusion currents), as shown in Figure 7b [139].

Moreover, the observed enhancement was reported to be thickness-dependent and significantly associated with a ‘photo-Dember’ electric field generated by the localized surface plasmon resonance (LSPR) [139,140]. Nonetheless, THz spectroscopy of CdTe indicates that THz absorption is analogous to that of ZnTe for frequencies below 1 THz but exhibits a significant rise above this threshold, with a pronounced absorption peak around 2.1 THz [141]. On the other hand, coherent THz emission from large HgTe NCs has also been reported, owing to its intraband absorption peak, which is tunable from 4 to 60 THz. For large, weakly self-doped HgTe nanocrystals, the broad THz absorption resonance has been attributed primarily to multiple intraband transitions of individual carriers between quantized states, while collective excitations were found to make a negligible contribution [142]. Nonetheless, tunable peak emission frequencies from HgTe NCs (0.4–0.8 THz) are found to be dependent on the incident angle of the linearly polarized fs pulses. The THz emission in these HgTe NCs is reported to be oriented in the direction normal to the HgTe film and induced by second-order nonlinear transient photocurrents arising from both the photogalvanic and photon drag effects [143]. Other reports suggest that the main carrier recombination process is attributed to direct energy transfer from the electronic transition to the ligand vibrational modes and to nonradiative recombination assisted by surface traps [142]. These findings open interesting perspectives for the use of HgTe NCs for the development of advanced THz optoelectronic emitters and detectors and for quantum engineering at THz frequencies.

In addition to CdTe and HgTe, the ability to engineer the bandgap of MCT (Hg_1−x_Cd_x_Te) heterostructures, from ~0.26 to 1.60 eV, enables it to cover a broad spectrum of IR wavelengths, making them highly attractive for IR detectors, THz lasers, and sensors. Numerous internal and external parameters influencing the bandgap, such as the Hg/Cd atomic ratio, quantum well thickness, electromagnetic (EM) fields, temperature, and hydrostatic pressure, can render these materials to behave as topological insulators (TIs), Dirac semimetals (DSs), or even as conventional narrow-gap semiconductors [144]. With an inverted band ordering, MCT has been explored for low-temperature non-resonant THz photoconductive detection, with its sensitivity enhanced close to the charge neutrality point and to the magnetic field-driven topological phase transition [145]. Additionally, electron heating by EM waves in MCT layers can be used to make uncooled THz/sub-THz detectors for application in imaging systems at room temperature. The sensitivity of such devices can be enhanced by controlling their initial layers, substrate properties, and antenna configurations [146].

**Figure 8:**
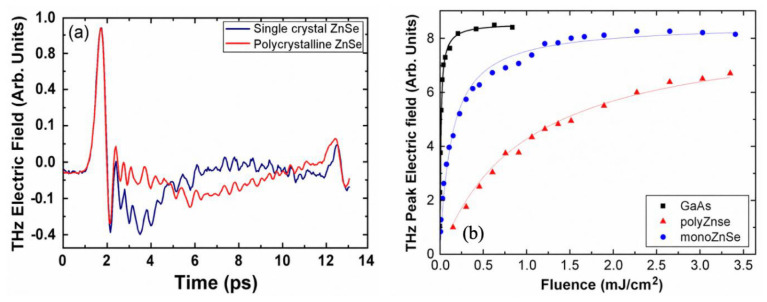
(**a**) Normalized THz waveforms emitted by the mono- and polycrystalline ZnSe LAPCAs. (**b**) Peak THz electric field as a function of fluence for GaAs, mono-, and polycrystalline ZnSe-based LAPCAs. Reproduced from Ref. [110] with permission from IEEE.

In the same II–VI family, zinc selenide (ZnSe) with its wider bandgap (~2.67 eV) is generally used as optical windows and lenses in the IR region. A few reports suggest its use for the development of high-power, large-aperture PCAs (LAPCAs) pumped with a Ti:Sapphire laser via standard one-photon absorption under the second harmonic (λ ~ 400 nm) and via two-photon absorption while using the fundamental harmonic wavelength (λ ~ 800 nm) [110]. Compared with GaAs, ZnSe exhibits a substantially higher dielectric-breakdown strength, enabling operation at larger applied bias fields. Its effective carrier mobility, however, remains strongly dependent on crystal quality, carrier concentration, and whether mono- or polycrystalline substrates are used. The THz emission response from LAPCAs made using the monocrystalline and polycrystalline ZnSe substrates has also been compared, as shown in Figure 8a. The performance of mono- and polycrystalline ZnSe LAPCAs (at λ ~ 400 nm) has been further compared with the GaAs LAPCAs (excited at λ ~ 800 nm) as shown in Figure 8b. The difference in THz peak electric fields between the three LAPCAs is reported to be due to differences in the photoexcited charge-carrier mobilities, resulting in three different saturation fluence regimes [110]. Table 2 provides a non-exhaustive comparison of different material properties, dominant THz-response mechanisms, and key advantages and limitations of representative group II–VI materials discussed in this section. In spite of their attractive optoelectronic properties and widespread use in THz photonics, some II–VI materials exhibit relatively lower carrier mobilities than leading III–V materials and are often constrained by multiphoton absorption saturation and restricted phase-matching. In addition, although materials such as ZnTe and CdTe are widely employed in THz emission and EO detection, their THz emission efficiencies generally remain lower than those reported for the most efficient III–V emitters [147,148,149].

### 3.3. Group IV Materials

Amongst many group IV materials, Si and Ge play a pivotal role in THz photonics due to their established processing infrastructure, CMOS compatibility, and tunable optoelectronic properties [50,51]. Table 3 lists some of these materials that bridge the gap between electronic compatibility and photonic functionality, presenting scalable and integrable platforms for next-generation THz sources and detectors. While indirect band gaps and low nonlinear optical coefficients affect THz radiation efficiencies, recent advancements in nanofabrication techniques, doping, and strain engineering help overcome these barriers.

Silicon (Si), being centrosymmetric in nature with an indirect bandgap (~1.12 eV), has been reported to show SHG and THz emission (via transient photocurrents driven by built-in surface/depletion fields) when fs pulses interact with the Si/SiO_2_ interface. Moreover, a direct relation between the resistivity of the p-Si (100) wafers and the polarity of their emitted THz radiation has also been established, whereas the azimuthal dependence of the THz emission from Si (001) exhibits a fourfold azimuthal symmetry. This is attributed to the bulk electric quadrupole and magnetic dipole of Si (001) [157]. Moreover, Si nanostructures have also garnered significant attention as efficient THz sources, highlighting a direct correlation with their geometry and depth. Compared with planar surfaces, black Si with needle-like nanostructures fabricated via reactive ion etching (RIE) exhibits a fourfold enhancement in THz emission [158]. In fact, Si exhibits a refractive index (~3.4) similar to the other semiconductors in the THz spectral range (0.1–10 THz), making it very useful in various THz optical setups [159]. In general, Si lenses have been used as substrate lenses to couple radiation from the emitter to the detector. Due to its low absorption and dielectric nature, high-resistivity float-zone Si exhibits the highest transparency in the THz range, making it a highly suitable component for all THz optical setups [160].

Moreover, some reports even suggest that using hyper-hemispherical Si lenses (instead of hemispherical lenses) reduces the resulting angular tilt when misplacing an antenna on the Si lens surface [161].

**Table 3:** Comparison of group IV materials investigated for THz generation, including their band gaps, carrier mobilities, dominant THz-emission mechanisms, and key advantages and limitations. The reported values are representative and may vary with material polytype, alloy composition, strain, doping, defect density, crystal quality, growth conditions, and device architecture.

Material	Bandgap (eV)	Electron/Hole Mobility (cm^2^ V^−1^ s^−1^)	Dominant THz Mechanism(s)	Key Advantages	Key Limitations	Refs.
**Si**	~1.2	~1350/~480	Surface-field emission, transient photocurrents	CMOS-compatible, low-cost fabrication	Indirect bandgap, weaker near-IR absorption	[157,158,162]
**SiC** **(4H/6H)**	~3.2	~500–1000/ ~100–115	Biased photoconductive emission	High breakdown strength, thermal conductivity	Wide indirect bandgap, requires multiphoton excitation	[163,164]
**Ge**	~0.6–0.8	~3900/~1900	Photo-Dember effect, surface depletion, EFIOR	High carrier mobilities, CMOS compatibility	Long native carrier lifetimes, growth-dependent THz response	[51,52,55]
**GeSn**	~0.3–0.6 (Sn dependent)	~520	Biased photoconductive current surge	Tunable band structure, strong absorption at 1.55–1.56 μm	High dark currents, defect formation at high Sn concentrations	[65,165,166]
**Diamond**	~5.5	~4500/~3800	Biased photoconductive current surge	Extremely high breakdown strength and thermal conductivity	Requires multiphoton or defect-assisted excitation, expensive	[167,168,169]

More recently, nonlinear optical processes such as two-photon absorption and third-harmonic generation in Si waveguides have also been reported to show efficient and compact on-chip integrated THz functionalities, especially when driven by the high-intensity fs laser pulses [170]. Among other group IV materials, polycrystalline diamond grown by CVD has been reported as an efficient LAPCA, particularly when implemented with an interdigitated electrode structure and illuminated by an excimer laser [167]. More recently, THz generation from nitrogen-doped single-crystal diamond LAPCAs under 400 nm femtosecond excitation has further demonstrated the strong influence of dopant concentration on photoconductivity and saturation behavior [168]. Despite promising results, with no signs of saturation as a function of the bias field, diamond LAPCAs remain insufficiently explored. Beyond their high cost and complex fabrication, this is largely due to diamond’s wide bandgap (~5.46 eV), which requires excitation via the fourth harmonic of an 800 nm Ti:Sapphire laser or through two-photon absorption under excimer laser illumination [163]. However, its THz performance remains strongly dependent on crystal quality, nitrogen concentration, grain structure, and carrier lifetimes. On the other hand, the generation of free-space THz pulses at lower frequencies has also been reported using another group IV material, namely 4H-SiC and 6H-SiC LAPCAs. At an excitation wavelength of 400 nm, the THz power radiated by a 6H-SiC PCA was reported to be 2.3 times larger than that from a ZnSe PCA under optimum conditions. This improvement was primarily attributed to the high thermal conductivity and dielectric strength of SiC, which enabled operation at elevated bias fields and optical fluences without the thermal limitations observed for ZnSe [164]. Nevertheless, the wide indirect band gaps of 4H- and 6H-SiC necessitate UV or multiphoton excitation and reduce the efficiency of transient photocarrier generation, with the resulting emission occurring predominantly in the low-THz frequency range.

Germanium (Ge), with smaller indirect (~0.66 eV) and direct (~0.80 eV) band gaps, tends to enhance ultrafast carrier dynamics when integrated with antenna designs, making it a promising platform for compact and broadband photoconductive THz emitters [51]. An in-depth analysis of the role of Ge as an efficient THz radiation source will be discussed in the subsequent section, where we elaborate on different deposition techniques, structural and optoelectronic properties, and device-level implications for THz emission using Ge. In addition, GeSn alloys have also emerged as promising group IV photoconductors for telecom-wavelength THz generation. A large-area emitter based on RPCVD-grown Ge_0.96_Sn_0.04_ on a Si substrate exhibited a room-temperature majority-carrier mobility of ~518 cm^2^ V^−1^ s^−1^, an absorption coefficient of ~7187 cm^−1^ at 1560 nm, and a carrier lifetime of ~435 ps. Moreover, it is reported to emit THz radiation extending beyond 2 THz with an SNR of approximately 40 dB when excited using a 1560 nm fs fiber laser [65]. Nevertheless, a substantial fraction of its radiated power remained below 400 GHz because of the comparatively long carrier lifetime, while high dark current, bias-field screening, and Joule heating limited further output scaling. These results demonstrate the potential of GeSn for CMOS-compatible and fiber-integrated THz systems while highlighting the need for improved carrier-lifetime and interface engineering.

### 3.4. 2D and Layered Materials

The advent of two-dimensional (2D) and layered materials has created new possibilities in THz science due to their unique physical properties, ultrathin thickness, and highly tunable electronic structure. Unlike bulk semiconductors, some of these 2D materials, such as graphene, layered black phosphorus (BP), and TMDCs (including MoS_2_, WS_2_, and WSe_2_), exhibit strong quantum confinement effects, resulting in enhanced light–matter interactions. Table 4 presents a non-exhaustive comparison of some 2D and layered materials investigated for THz emission, highlighting their band gaps, carrier mobilities, dominant emission mechanisms, and key advantages and limitations. Since 2004, graphene has emerged as a highly attractive ultrathin 2D material exhibiting semi-metallic behavior and exceptional intrinsic carrier mobility [171], thereby opening the door to numerous applications such as broadband optical modulators [172], ultrafast photodetectors [173], mode-locking ultrafast lasers [174], tunable dual-frequency THz sensors [175], and graphene Tera-FETs [176]. Of particular interest is graphene’s optical absorption, which involves both intraband and interband transitions with relative contributions that vary across the electromagnetic spectrum. While interband transitions dominate the optical and NIR wavelengths, the intraband transitions take place upon interaction with low-energy photons (far-IR and THz). The optical response of graphene can be tuned by altering its Fermi level, typically done via electrical means, optical stimulus, or chemical doping [177].

In addition, graphene can exhibit room-temperature carrier mobilities ranging from approximately 10^3^–10^4^ cm^2^V^−1^ s^−1^ in conventional or processed devices to above 10^5^ cm^2^V^−1^ s^−1^ in high-quality encapsulated structures, with nearly symmetric ambipolar carrier transport [178]. The high carrier mobility with low electrical resistance and ultrafast carrier-relaxation dynamics make graphene an attractive platform for broadband THz generation [179]. Nevertheless, these exceptional transport properties do not directly translate into strong free-space THz emission because the atomically thin active volume limits the optical interaction and radiated power. In general, efficient graphene emitters commonly rely on symmetry-breaking interfaces, antenna coupling, multilayer configurations, or plasmonic field enhancement [180].

While graphene is a near-zero bandgap 2D material, transition metal dichalcogenides (such as MoS_2_, MoSe_2_, WS_2_, and WSe_2_) form a family of 2D semiconductors with direct bandgaps that can be tuned from the visible to the near-infrared as their thickness is reduced to a few monolayers [181]. This makes them useful as THz emitters when excited by ultrafast fs laser pulses via surface nonlinear optical polarization and surface field-induced photocurrent. With a repeated stack of X-M-X layers held by van der Waals (VDW) interactions, where M is a transition metal and X is a chalcogen (S, Se, or Te), semiconducting TMDCs with a large on/off ratio, ultrafast speed, and high photoresponsivity have been reported to be useful for various applications in THz generation devices, modulators, THz shielding, and photodetectors [63,182,183].

**Table 4:** A non-exhaustive comparison list of some 2D and layered materials used for THz generation, including their band gaps, carrier mobilities, dominant THz-emission mechanisms, and key advantages and limitations. The reported values are representative and may vary with layer thickness, defect density, substrate type, and device architecture.

Material	Bandgap (eV)	Electron/Hole Mobility (cm^2^ V^−1^ s^−1^)	Dominant THz Mechanism(s)	Key Advantages	Key Limitations	Refs.
**Graphene**	0 (semi-metal)	~10^3^–10^5^	Photon drag currents, built-in-field-driven photocurrents	Ultrafast carrier dynamics, broadband tunable response	Weak free-space emission, substrate-dependent performance	[178,179,180]
**MoS_2_**	~1.2–1.9	~470/~480	Surface optical rectification, surface photocurrent surge	Strong light–matter interaction, scalable synthesis	Layer thickness-dependent mobility, limited active volume	[184,185,186]
**WS_2_**	~1.3–2.1	~10–100	Surface-field transient drift currents, linear/circular optical rectification	Strong excitonic absorption, scalable synthesis	Sensitive to defects, weak emission with monolayers	[187,188,189]
**MoSe_2_**	~1.1–1.6	~50–160	Surface depletion field, surface optical rectification	Strong excitonic absorption, thickness-dependent tunability	Layer thickness-dependent mobility, limited emitter literature	[190,191,192,193]
**WSe_2_**	~1.2–1.6	~30/~80–350	Surface depletion field, in-plane/out-of-plain drift current	Ambipolar transport, Strong excitonic and spin–valley response	Thickness dependence, limited active volume	[182,194,195,196]
**Black Phosphorus (BP)**	~0.3–1.7	~100–1000/~200–650	Anisotropic surface photogalvanic current	Thickness tunable bandgap, Compatible with NIR	Environmental instability, rapid photo-oxidation	[197,198,199,200]

Among these materials, MoS_2_ provides an example of the strong influence of dimensionality and surface symmetry on THz emission from layered semiconductors. Bulk 2H-MoS_2_ possesses an indirect bandgap of ~1.29 eV, whereas reducing the material to a monolayer produces a direct transition near ~1.90 eV [186]. Polarization- and azimuth-dependent THz emission measurements have revealed competing contributions from surface optical rectification, enabled by inversion-symmetry breaking at the surface, and transient photocurrent acceleration within the surface depletion field [184]. More recently, Nb doping of atomically thin MoS_2_ has been reported to show an enhancement in the emitted THz field, demonstrating that the surface electrostatic potential, charge screening, and defect landscape can be engineered to control the amplitude and polarity of the THz emission [105]. Nevertheless, the transport and THz-emission characteristics of MoS_2_ remain strongly dependent on layer thickness, substrate and dielectric environment, contact resistance, doping, and structural defects [185].

In layered WS_2_ crystals, THz emission has been attributed predominantly to the acceleration of photoexcited carriers by the out-of-plane surface depletion field [189]. In monolayer WS_2_, however, the absence of inversion symmetry permits second-order nonlinear processes, while polarization-resolved measurements have demonstrated THz emission from in-plane dipoles through both the linear and circular optical rectification [187]. Furthermore, a distinct mechanism emerges when monolayer WS_2_ is combined with MoS_2_ in a type-II van der Waals heterostructure, where ultrafast charge separation across the sub-nm interface generates a transient interlayer current and the corresponding THz radiation [201]. These studies demonstrate that the dominant THz-emission mechanism in WS_2_ is strongly architecture-dependent, ranging from surface-field-driven drift currents in layered crystals to optical rectification in non-centrosymmetric monolayers and interlayer charge-transfer currents in heterostructures.

**Figure 9:**
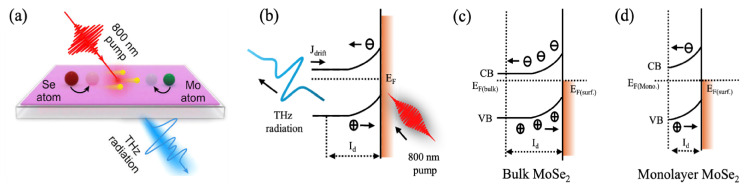
Physical THz radiation mechanism for TMDCs, including (**a**) optical rectification [16], and (**b**) surface depletion field-induced photocurrent effect in (**c**) bulk and (**d**) monolayer MoSe_2_. Figure 10a reproduced from Ref. [16] under CC BY 4.0.

Figure 9a illustrates the THz emission mechanism from MoSe_2_, involving absorption via resonant optical rectification (hν ≥ E_g_), which induces charge-carrier motion and results in instantaneous polarization. This rapid change in polarization, occurring on an fs timescale, is associated with variations in the charge density around the Mo and Se atoms at a bond length scale [16]. A second contribution arises from the built-in electric field within the surface depletion region, which results from surface Fermi-level pinning and the associated band bending, as illustrated in Figure 9b. Furthermore, Figure 9c,d show that this band bending induces a drift current and subsequent THz radiation. The broader surface-charge region in bulk than that of monolayer MoSe_2_ influences the surface electric field of both materials [193]. In MoSe_2_, measurements on bulk and monolayer samples have revealed contributions from both surface optical rectification and surface-field-driven transient photocurrents, with the photocurrent contribution dominating under the investigated excitation conditions [193].

Layered WSe_2_ exhibits a similarly architecture-dependent THz response. In bulk or thick layered crystals, the initial THz pulse is attributed predominantly to an out-of-plane transient drift current generated by the acceleration of photoexcited carriers within the surface depletion field [202]. In few-layer WSe_2_, however, polarization-resolved measurements revealed the simultaneous generation of an in-plane shift current and an out-of-plane drift current. The phase difference between the corresponding THz-field components produces elliptically polarized radiation whose ellipticity can be controlled through the polarization of the incident fs pulses [196]. The initial current-driven emission may also be followed by oscillatory radiation associated with IR-active interlayer shear and breathing phonon modes [182]. These results demonstrate that the THz emission from WSe_2_ is governed by the combined influence of layer number, surface electrostatics, inversion symmetry, excitonic excitation, and lattice dynamics.

Black phosphorus (BP) represents a distinct class of anisotropic layered semiconductor whose direct bandgap varies strongly with thickness, from ~0.35 eV (in bulk) to ~1.73 eV (in monolayer limit) [197]. Its puckered crystal structure gives rise to strongly direction-dependent optical absorption and carrier transport, with higher mobility generally observed along the armchair direction [198]. However, free-space THz emission from bulk BP has revealed an anisotropic second-order surface photocurrent that can be described by the photogalvanic effect, enabled by inversion-symmetry breaking at the crystal surface [199]. Moreover, multilayer BP has also been incorporated into hBN-encapsulated PCAs operating under 780 and 1560 nm fs excitation, where the bias-induced transient photocurrent was used to model the expected THz output [200]. Despite its tunable bandgap, high mobility, and intrinsic anisotropy, the practical use of BP remains restricted by rapid photo-oxidation under ambient conditions, necessitating careful handling and encapsulation [203].

Even though TMDCs have lower carrier mobilities as compared to graphene, the strong excitonic resonances and spin–valley locking under circularly polarized light offer novel opportunities for polarization-sensitive and valleytronic THz modulation using these materials [204]. Overall, 2D and layered materials tend to offer broad prospects for the fabrication and engineering of VDW heterostructures while enabling significant broadband electrical tuning of THz radiation. However, their atomically thin nature inherently limits light–matter interaction and optical absorption, often resulting in lower THz emission efficiencies than established bulk semiconductor platforms. Furthermore, large-area synthesis, device reproducibility, and heterostructure engineering continue to impede the widespread technological deployment of these materials.

### 3.5. Topological Insulators

Topological insulators (TIs) are a novel category of quantum materials attracting significant interest in recent years owing to their distinctive electronic structures, characterized by an insulating bulk and gapless, spin-momentum-locked conducting surface states protected by time-reversal symmetry [205]. These properties, along with the presence of chiral spin structures, make TIs a material of choice for THz emission through ultrafast photoexcitation mechanisms. In general, the THz emission from 3D-TIs with highly spin-polarizable material layers is primarily due to the conversion of ultrafast spin currents to charge currents via the inverse Edelstein effect at the surface/interface and the inverse spin Hall effect in the bulk [206]. Efficient THz emission at room temperature from the prototypical 3D-TI, n-doped bismuth selenide (Bi_2_Se_3_), has been attributed to multiple, experimentally distinguishable mechanisms. Polarization-resolved measurements identified the contributions from transient photocurrents driven by the surface depletion field near the TI surface and from nonlinear optical rectification, reflecting the bulk electronic properties [207]. Subsequent ultrabroadband THz-emission spectroscopy resolved an additional ultrafast surface-shift current arising from photoinduced charge displacement along the Se–Bi bonds, together with a slower drift current generated by bulk carriers within the surface space-charge field [208]. These findings indicate that the relative contributions of surface and bulk photocurrents depend strongly on doping, surface band bending, excitation conditions, and experimental bandwidth. Moreover, THz emission from Bi_2_Se_3_/ferromagnetic Co heterostructures has been reported to outperform that of either Bi_2_Se_3_ or Co individually [209], due to ultrafast spin-to-charge conversion mediated by the topological surface states. Temperature-dependent measurements further showed that THz emission from pure Bi_2_Se_3_ increases upon cooling, consistent with its enhanced carrier mobility, whereas the extracted spin-to-charge-conversion component from the Bi_2_Se_3_/Co heterostructure remains nearly temperature-independent between 10 and 300 K, indicating a temperature-insensitive inverse Edelstein conversion efficiency [210].

**Figure 10:**
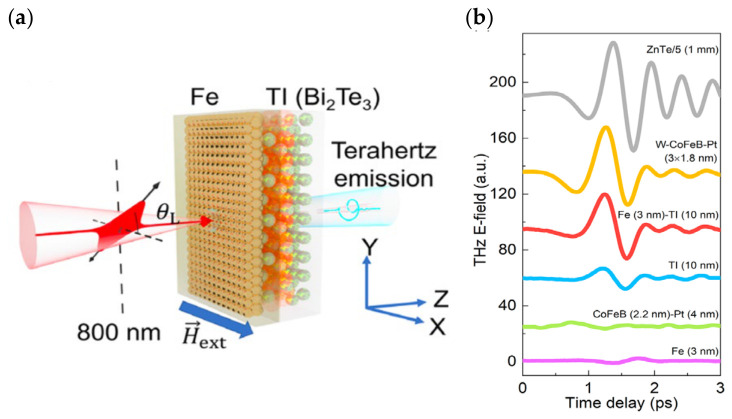
(**a**) Schematic diagram of the TI–Fe heterostructures driven by fs laser pulses. (**b**) Typical THz temporal waveforms generated from Fe (3 nm), CoFeB (2.2 nm)–Pt (4 nm), TI (10 nm), TI (10 nm)–Fe (3 nm), W (1.8 nm)–CoFeB (1.8 nm)-Pt (1.8 nm), and ZnTe (1 mm) thin films, respectively. Reproduced from Ref. [213] under CC BY 4.0.

Other 3D-TIs, particularly Bi_2_Te_3_, exhibit strong nonlinear surface photocurrents and polarization-dependent THz emission. In Bi_2_Te_3_ nanofilms, linear and circular photogalvanic effects generate helicity-independent and helicity-dependent photocurrents, respectively, enabling control over the polarization, ellipticity, and chirality of the emitted THz radiation [211,212]. Moreover, as shown in Figure 10a, Bi_2_Te_3_/Fe heterostructures have also been reported to radiate high-quality elliptically polarized THz waves with an extremely high THz emission efficiency, compared to that from W (1.8 nm)–CoFeB (1.8 nm)–Pt (1.8 nm) (Figure 10b) [213]. This is primarily due to the presence of spin-to-charge conversion at the TI-Fe interface and the excited linear photogalvanic effect at the surfaces of Bi_2_Te_3_ arising from inversion symmetry breaking. The magnetic control method, along with optical techniques, allows polarization modulation of THz radiation at a sub-ps time scale [213]. Under equivalent excitation conditions, the emitted peak-to-peak THz field reached approximately 67.5% of that produced by a benchmark W/CoFeB/Pt trilayer, demonstrating comparable rather than superior emission efficiency [213]. Similarly, intense spintronic THz emission has also been reported from topologically nontrivial semimetal Bi_1−x_Sb_x_ films. Such THz radiation is significantly stronger than that observed in Pt and Bi_2_Se_3_, further highlighting the potential of Bi_1−x_Sb_x_ for spintronic applications [107].

In Sb_2_Te_3_, polarization-resolved THz-emission spectroscopy has enabled the separation of distinct ultrafast photocurrent contributions. The observed emission contains helicity-independent linear photogalvanic, helicity-dependent circular photogalvanic, and photothermoelectric components, with the CPGE contribution arising predominantly from the spin-momentum-locked topological surface states. Moreover, it has been reported that a reversal of the optical helicity effectively reverses the corresponding photocurrent and THz-field polarity. Nonetheless, the relative strengths of these mechanisms depend strongly on film thickness, temperature, crystal orientation, surface morphology, and structural strain, demonstrating the potential for all-optical and mechanically tunable control of THz emission [214,215]. Table 5 presents a non-exhaustive list of some of the TIs with their dominant THz mechanisms and key advantages and limitations. With ongoing advancements in material science, surface engineering, and heterostructure design, these efficient TI-heterostructure-based THz radiation sources hold promise for next-generation ultrafast optoelectronic devices and quantum THz systems, including polarization-sensitive THz sensing, imaging, and information encryption. Nevertheless, the THz response of TIs is often highly sensitive to crystal quality, stoichiometry, and defect-induced bulk conduction, making it difficult to isolate and fully exploit the topological surface states that underpin many of their unique optoelectronic properties.

### 3.6. Weyl Semimetals

**Figure 11:**
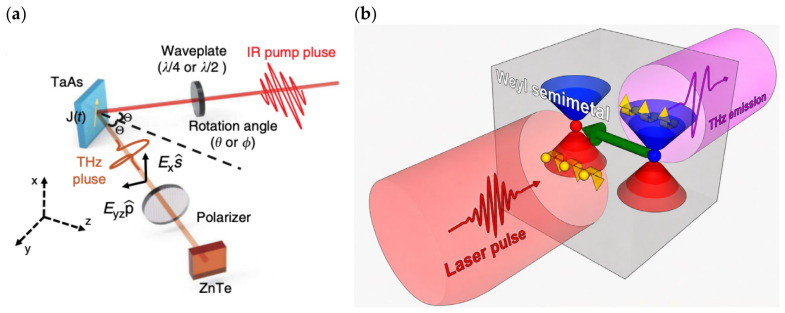
Schematics of the THz emission spectroscopy setup using (**a**) a single crystal of TaAs [64] and (**b**) a single-layer Weyl semimetal where the magnetization and anomalous Hall effect (AHE) influence the efficiency of the THz radiation [226]. Reproduced from Refs. [64,226] under CC BY 4.0.

Weyl semimetals (WSMs) are characterized by their distinctive topological electronic properties, which arise from the presence of chiral quasiparticles near the Weyl nodes. Previous works highlight the emergence of various fascinating optoelectronic responses in WSMs associated with the Berry curvature of the topological bands, including spin-polarized photovoltaic currents, the photoinduced anomalous Hall effect, and quantized photocurrents. In addition, WSMs exhibit ultrafast charge separation via shift-current mechanisms when subjected to THz excitation. For mid-IR experiments, WSMs, such as tantalum arsenide (TaAs), exhibit a dominant helicity-dependent direct photocurrent arising from the circular photogalvanic effect, associated with optical transitions between tilted anisotropic Weyl cones and massive bulk bands, as shown in Figure 11a [64]. Moreover, the emitted THz radiation exhibits controllable polarization, making TaAs a unique “chiral photon source” enabling novel quantum optoelectronic devices [64]. Recent studies have also introduced new design concepts that leverage topological effects in spin-based THz emitters, providing further insight into Weyl physics. In particular, a single layer of magnetic WSM, such as Co_2_MnGa, has been reported to exhibit strong and efficient THz emission, with a large anomalous Hall effect influenced by its Weyl semi-metallic nature and chemical ordering in full Heusler crystal structures, as shown in Figure 11b [226]. In this context, magnetic WSMs, including Co_2_MnGa and Co_2_MnAl, have been known to exhibit THz spin-current emission efficiencies and relaxation times (170 fs and 100 fs, respectively) comparable to those of conventional 3D transition-metal ferromagnets such as Fe (90 fs). This performance, combined with topologically driven spin dynamics, underscores the potential of magnetic Weyl compounds for ultrafast THz spintronic applications [227]. Along with the TIs, Table 5 also lists some of the commonly known WSMs that are reported for THz emission, along with their dominant THz mechanisms and key advantages and limitations. Despite these promising results, WSM-based THz emitters remain at a relatively early stage of technological maturity. Moreover, their performance is often highly sensitive to crystal quality and crystallographic orientation, while the underlying emission mechanisms continue to be actively explored.

## 4. Germanium: A Promising Material for THz

Broadly, Ge has also been investigated for a variety of photonic applications, including low-loss waveguides [229,230], modulators in mid-IR photonic devices [231,232], optical gain media [233,234], solar cells [235,236], and photodetectors [237,238]. However, under fs optical excitation, Ge has re-emerged as a compelling platform for broadband THz emission and nonlinear THz–matter interaction studies, generating THz transients via ultrafast photocurrent mechanisms (e.g., surface-field/surge-current and photo-Dember-type contributions) [54], and electric field-induced optical rectification [56]. In addition, Ge-based PCAs have demonstrated smooth, gapless broadband THz spectra extending up to ~70 THz [67]. This highlights Ge as a material with strong THz fields and non-Drude transient ultrafast carrier dynamics [239]. Moreover, the pronounced transient field-dependent scattering and impact-ionization effects directly shape the THz conductivity and absorption features, motivating extensive experimental and atomistic modeling efforts in recent years [55,240]. From a technology perspective, these developments position Ge as an attractive bridge between high-performance THz emitters and fabrication routes compatible with large-area deposition and heterogeneous integration, making it relevant both as a high-field nonlinear THz material and as a practical emitter medium for emerging THz systems.

**Figure 12:**
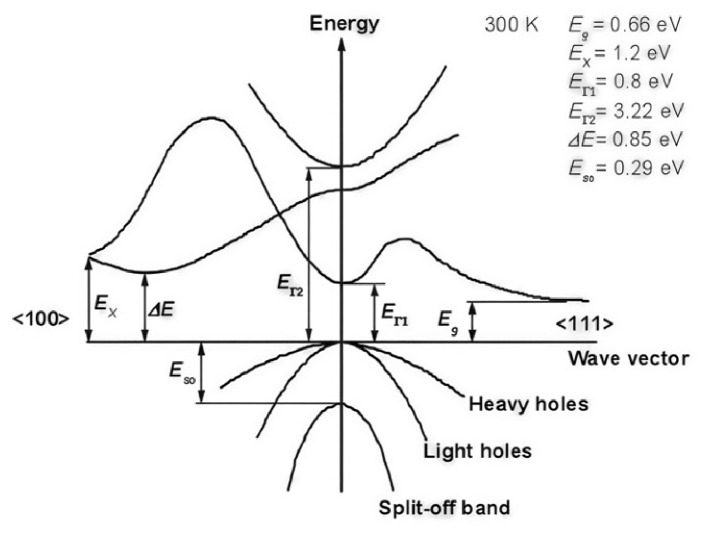
Energy-wave vector diagram of Ge showing the different valleys in the conduction and valence bands and associated energy gap transitions. Reproduced from Ref. [241] under CC BY-NC-SA 3.0.

Figure 12 shows the energy-momentum (E-k) diagram of Ge with different valleys in the conduction and valence bands [241]. Although Ge has historically been overshadowed by the optoelectronic properties of III–V and II–VI materials, its high carrier mobility, absence of polar phonons, narrow indirect bandgap ($E_{g}^{L}\sim$0.66 eV at 300 K), and strain- or alloy-induced direct bandgap ($E_{g}^{\Gamma }\sim$0.80 eV) transitions [242,243], have positioned it as a key candidate for efficient and broadband THz emission [244]. In particular, Ge exhibits high electron (~3900 cm^2^V^−1^ s^−1^) and hole (~1900 cm^2^V^−1^ s^−1^) mobilities, as well as long minority carrier diffusion lengths, which enable efficient charge transport and THz radiation under ultrafast fs excitation [51]. Moreover, the optical absorption of Ge can be enhanced from 63% (on flat-surface wafers) to as high as 99% over the whole 300–1600 nm spectral range when its surface is nanostructured using an SF_6_-based reactive ion etching process [245]. This advantageously leads to a graded refractive index effect extending into the NIR regime. Similar enhancement and tunability in refractive index have also been reported from sputtered Ge films in the mid- and long-wave IR bands [246]. In addition, the relatively large dielectric constant (ε~16) at RT [247], amplifies the internal space-charge fields generated upon photoexcitation. Collectively, these space-charge fields and tunable optical properties significantly influence the design of optoelectronic devices operating in the mid- and long-wave IR regions, while also playing a key role in the acceleration of photoexcited charge carriers responsible for efficient THz radiation.

Numerous deposition techniques have been employed for the growth of Ge thin films, including molecular beam epitaxy (MBE) [248], RF-magnetron sputtering [111], chemical vapor deposition (CVD) [249], pulsed laser deposition (PLD) [250], and thermal evaporation [251]. Most of these methods enable precise control over film thickness, structure, morphology, doping concentration, crystalline orientation, and defect density. These microstructural parameters critically influence the optoelectronic properties of the grown Ge films, and in particular the temporal profile and intensity of the emitted THz pulse. In addition to growth techniques, factors such as strain engineering, doping, and alloying with Sn (GeSn) provide additional control over Ge’s band structure, enabling transitions toward a direct bandgap and thereby enhancing carrier injection efficiency and radiative recombination rates under femtosecond laser excitation. The following subsections explore the fundamental and applied aspects of THz emission in Ge, beginning with an analysis of its band structure and strain-induced direct transitions, followed by nonlinear charge-carrier dynamics with an emphasis on the role of dopants, and finally underlying the impact of fabrication techniques. We conclude with the performance benchmarks for Ge-based THz emitters in optoelectronic systems and a few emerging applications.

### 4.1. Band Structure and Strain-Induced Direct Transitions

**Figure 13:**
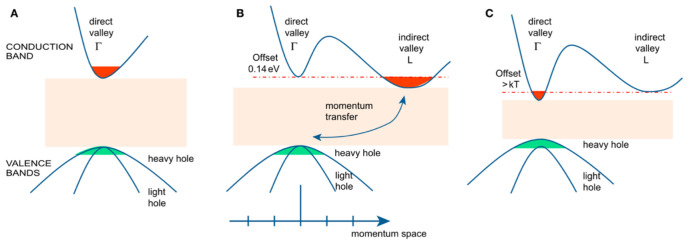
Band structures in momentum space for (**A**) a direct bandgap semiconductor, (**B**) unstrained Ge, and (**C**) strain-induced Ge. Reproduced from Ref. [252] under CC BY 4.0.

A critical determinant of THz emission efficiency in Ge is the subtle interplay between its band structure and the ultrafast carrier dynamics under various ultrafast excitation and strain conditions. Compared to a direct bandgap semiconductor shown in Figure 13A, intrinsic unstrained Ge (Figure 13B), with an indirect bandgap ($E_{g}^{L}\sim$0.66 eV @ 300 K), has its conduction band minima located at the L-point of the Brillouin zone, while the direct Γ-point lies at a higher energy level ($E_{g}^{\Gamma }\sim$0.80 eV). The energy separation (∆E~134 meV) between Γ and the L valley introduces a bottleneck in the generation and recombination of photoexcited carriers, thereby limiting efficient radiative emission pathways in the absence of external perturbations. However, this limitation can be mitigated either by introducing biaxial tensile strain or alloying with Sn, which reduces the energy separation (${∆E}_{\Gamma -L}$) and drives the system toward a direct bandgap regime under sufficient strain levels (typically > 1.8%), as shown in Figure 13C [252].

**Figure 14:**
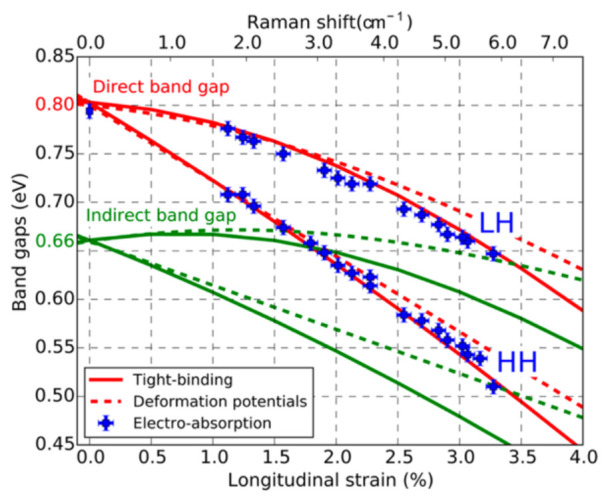
Band gaps measured by electroabsorption spectroscopy (blue squares) as a function of longitudinal strain measured by Raman spectroscopy. The experimental points are compared to theoretical bandgap values computed with deformation potentials (dashed red line) and with a tight-binding model (solid lines). The upper and lower curves correspond to the transition with the light-hole (LH) and the heavy-hole (HH) band, respectively. Reproduced from Ref. [253] © 2016 American Chemical Society.

In Ge, the Γ-valley undergoes a more pronounced downward energy shift than the L-valley, rendering it energetically favorable for photoexcited electrons to populate the Γ-valley under tensile strain. This redistribution of carriers into a valley with lower effective mass enhances mobility and accelerates intraband relaxation, thereby increasing the transient photocurrent (J(t)), which ultimately gives rise to broadband THz radiation. Under strain conditions, the effective carrier mobilities exceed those of unstrained films, further highlighting Ge as a promising low-bandgap material for strong THz emission. As shown in Figure 14, the direct bandgap of Ge as a function of [100] uniaxial strain up to 3.3% has been experimentally determined using electroabsorption spectroscopy, revealing its nonlinear dependence on the applied longitudinal strain [253]. These results are supported by theoretical models based on second-order deformation potentials within the model-solid theory, as well as tight-binding calculations derived from ab initio data that accurately reproduce the band structure of Ge under arbitrary strains in the ± 5% range [254,255]. Time-resolved pump-probe studies have further confirmed that the strain influences both the amplitude and temporal profile of the emitted THz waveform, often leading to broader and more coherent spectra. Overall, these findings demonstrate that strain engineering in Ge can serve as a powerful tuning parameter for optimizing both band alignment and carrier transport pathways, positioning Ge as a highly promising material for efficient THz generation.

### 4.2. Nonlinear Charge-Carrier Dynamics in Ge

**Figure 15:**
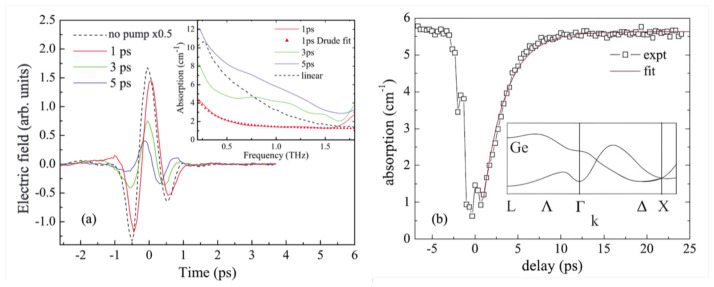
(**a**) The incident (black dotted line) and transmitted THz probe-pulse waveforms for Ge for 1 ps, 3 ps, and 5 ps probe delays, respectively. The inset shows the corresponding absorption spectra, the linear absorption spectrum (black dotted line), and a fit to the sum of two Drude-type components at 1 ps (red triangles). (**b**) Spectrally averaged probe-pulse absorption as a function of the THz probe delay for Ge. The inset shows the schematic illustration of the conduction-band structure. Reproduced from Ref. [239] © 2010 American Physical Society.

In many semiconductors, including Ge, intense single-cycle THz electric fields drive the photoexcited charge carriers far from their equilibrium, where the nonlinear response is commonly observed as a reduction in free-carrier absorption, i.e., the sample becomes more transparent to THz as the electric field increases. This is because the carriers are driven into states with lower effective mobility due to enhanced scattering, carrier heating, field-driven velocity saturation, and intervalley redistribution toward higher-effective-mass valleys, ultimately reducing the Drude-like conductivity at THz frequencies. This pulsed laser bleaching-dominated response is well documented in various materials such as Si, GaAs, Ge, InAs, InSb, HgCdTe, and InAs_0.4_Sb_0.6_ [256]. However, in contrast to many of these materials, Ge exhibits uniquely complex ultrafast carrier dynamics under intense THz excitation. As shown in Figure 15a, the time-resolved THz pump–THz probe measurements (with variable delays of 1, 3, and 5 ps in between pump and probe pulses) reveal a strong saturation of free-carrier absorption in n-type Ge at 300 K when exposed to single-cycle THz pulses with intensities up to 150 MW/cm^2^ [239]. In this regime, non-Drude-type absorption spectra observed in Ge at short probe delays (3 and 5 ps) indicate clear deviations from conventional free-carrier behavior, as evident by the THz absorption spectra shown in the inset of Figure 15a. Moreover, experiments performed at varying THz pulse energies show that, at intermediate THz excitation levels, a slight increase in absorption can occur in bulk Ge. This contrasts with the monotonic decrease in absorption typically observed in other semiconductors such as GaAs and Si, where side-valley mobilities are lower [239].

To further probe this ultrafast transient carrier redistribution within the conduction band of Ge, nonlinear THz absorption measurements were also conducted without the THz pump beam. As shown in Figure 15b [239], the spectrally averaged probe pulse excites carriers into the conduction band within ~1 ps, leading to a transient decrease in the free-carrier absorption coefficient, which can be described using the Drude model [257]. However, after ~5 ps, the system relaxes back toward equilibrium, and the cycle repeats: carriers absorb energy, redistribute among different conduction band valleys, and subsequently relax. This anomalous THz-induced nonlinear carrier dynamics in Ge is primarily attributed to a non-equilibrium multivalley carrier distribution, where Ge features an intermediate energy Γ-valley with significantly higher mobility (due to its smaller effective mass) [258] than that of the initially occupied L-valley, thereby contributing disproportionately to the THz conductivity spectrum. Although the Γ-valley population in Ge is typically negligible under equilibrium conditions, owing to its much lower density of states (~50 times smaller than that of the L-valley), its influence becomes pronounced under strong non-equilibrium excitation, as revealed in THz pump-probe measurements [239,258]. The inset of Figure 15b illustrates a simplified conduction band structure of Ge. Complementing the experimental observations, atomistic Monte Carlo simulations indicate that the total scattering rate is not constant, as assumed in the Drude model, but instead depends strongly on the applied peak THz field strength. These simulations, which incorporate acoustic, intervalley, Coulomb, and impact ionization scattering mechanisms, are in good agreement with the experimentally observed absorption spectra over a wide range of THz field strengths. Furthermore, the threshold field for the onset of impact ionization in Ge is found to be ~12 kV/m, leading to the generation of additional free carriers [240]. This enhances the Coulomb scattering at higher THz field strengths while significantly influencing the resulting carrier conductivity. These reported findings collectively outline a comprehensive picture of the complex interplay between intense THz fields and nonlinear carrier dynamics in Ge.

### 4.3. Effect of Doping and Ion Implantation

The doping and ion implantation of Ge have been proposed as among the most effective methods for controlling THz radiation by influencing photoexcited carrier dynamics, internal electric field profiles, and inherent scattering mechanisms. The magnitude and characteristic polarity of the THz transients have been reported to vary with both the type and concentration of the dopant. Even though the polarity of the emitted THz signal remains unchanged, n-type Ge samples are reported to exhibit a larger THz transient amplitude compared to the p-type ones, often indicating that the photocurrent surge driven by the built-in surface electric field is not the primary mechanism for plasma oscillation excitation. Instead, the photo-Dember effect, arising from the differing diffusion rates of photoexcited electrons and holes (μ_e_ >> μ_h_), is considered to be the dominant mechanism. In addition, the dependence of THz radiation spectra on the crystal doping levels has been interpreted to suggest that the primary mechanism responsible for THz emission in doped Ge crystals is cold plasma oscillation, where a maximum THz transient amplitude is observed at extrinsic carrier densities of ~10^17^ cm^−3^ for n-type and ~10^16^ cm^−3^ for p-type Ge crystals [54,56]. Furthermore, the carrier lifetime in Ge (ranging from 30 ns to 500 µs for bulk) is highly dependent on its resistivity and excitation level, owing to the ultrafast filling of recombination centers. For heavily doped materials, bulk recombination dominates, whereas trap-assisted Auger processes or interband Auger recombination significantly influence carrier lifetimes for dopant concentrations above 10^18^ cm^−3^ [259]. On the other hand, ion implantation has been employed to drastically reduce carrier lifetimes by introducing deep trap recombination states. This technique has been successfully used to overcome the relatively long carrier lifetimes (several µs) in intrinsic Ge, which are often limited by the laser pulse duration. For instance, O^+^ ion implantation into Ge films deposited on sapphire substrates has been reported to reduce the photocarrier lifetime down to 600 fs [66].

**Figure 16:**
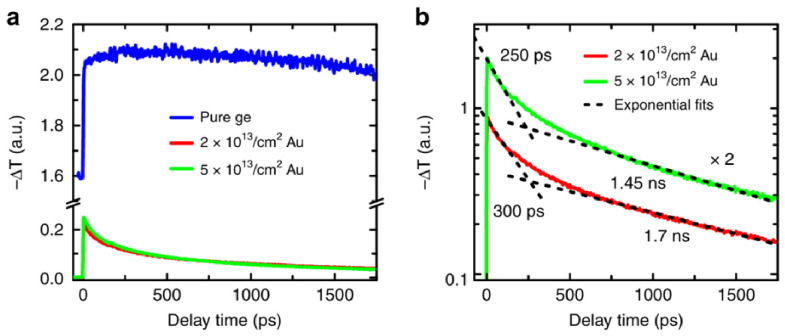
(**a**) Pump-induced change in the THz transmission as a function of the pump-probe delay time for pure and Au-implanted Ge. (**b**) Bi-exponential fits of the decay dynamics for the Ge:Au samples. Reproduced from Ref. [67] under CC BY 4.0.

More recently, Au implantation in Ge wafers has further reduced carrier lifetime to less than 2 ns, as shown in Figure 16a,b, by introducing deep acceptor levels that act as trapping and recombination centers [67]. The strategic use of doping and ion implantation makes Ge a highly promising material for THz applications. The ability to achieve ultrashort carrier lifetimes through implantation enables ultrabroadband THz emission, with reported bandwidths of up to 70 THz when pumped at 1100 nm and up to 50 THz when pumped at the telecom wavelength of 1550 nm [67]. This capability, combined with Ge’s high carrier mobility, its absorption edge compatibility with compact fiber lasers, and process compatibility with Si CMOS technology, positions Ge-based THz devices as strong candidates for next-generation THz technologies with continuous spectral coverage and scalable integration.

### 4.4. Fabrication Methods of Different Ge Structures

In general, the fabrication methods commonly used to deposit Ge in the form of thin films (amorphous, nanocrystalline, or polycrystalline) as well as various nanostructures (including nanowires, nano-bullets, nano-cones, etc.) significantly influence their optoelectronic properties, crystallinity, defect density, carrier mobility, and ultimately their THz radiation characteristics. Among the various thin film deposition techniques mentioned earlier, RF and DC magnetron sputtering is a highly controllable and scalable method for depositing Ge films with tailored morphology, thickness, and crystallinity. Notably, sputtering at low deposition temperatures (T_d_ < 300 °C) typically yields amorphous (a-Ge) films with limited carrier mobility [260], whereas higher T_d_ values (≥400 °C) or post-annealing treatments are known to produce films with high crystallinity (larger crystallites), improved carrier mobility, and extended carrier diffusion lengths [261]. Indeed, THz emission from ultrathin a-Ge layers (10–150 nm) deposited on glass and Au substrates via the RF-magnetron sputtering technique has been reported to be dependent on film thickness and the nature of the underlying substrate. With both the photo-Dember effect and the surface depletion field as the underlying THz emission mechanisms, a sevenfold enhancement of the THz electric field is observed from 25 nm-thick amorphous Ge films deposited on Au, compared to those deposited on glass substrates [111]. Nonetheless, a nontrivial correlation between the emitted THz amplitude and pump light absorption has been reported to arise from the fact that surface-related THz emission occurs within the Schottky depletion region at the Ge/Au interface, whose extent is close to ~25 nm, thereby yielding the maximum THz amplitude [111]. In contrast to THz emission from amorphous Ge films, we have demonstrated efficient THz emission from optimized and highly oriented-(111) n-doped polycrystalline Ge films deposited on sapphire substrates by means of RF-magnetron sputtering [52].

**Figure 17:**
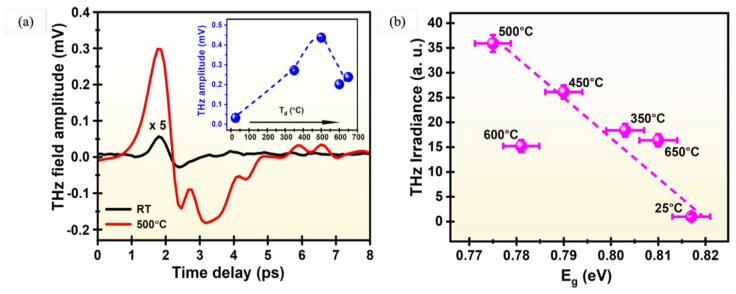
(**a**) THz spectra emitted by 570 nm-thick polycrystalline Ge films deposited on sapphire at RT (25 °C) and 500 °C. The inset shows the dependence of the emitted THz field amplitude on deposition temperature. (**b**) Total THz irradiance integrated over the measured spectral range as a function of the effective direct bandgap of the polycrystalline sputtered Ge thin films. Adapted from Ref. [52] under CC BY-NC-ND 4.0.

As shown in Figure 17a, the optimal sputtering conditions (T_d_ = 500 °C and thickness of 570 nm) were identified, yielding polycrystalline Ge films emitting the highest THz field amplitude with an amplification factor of ~26 (inset of Figure 17a) compared to a-Ge films deposited at T_d_ = 25 °C. This enhancement is attributed to the combination of optimized structural and optoelectronic characteristics, including a strong (111) preferential orientation, a narrowed effective direct bandgap (~0.77 eV), and the lowest electrical resistivity (of ~0.04 Ω·cm) [262]. Notably, despite the relatively limited variation in bandgap, the THz total irradiance of Ge thin films deposited at T_d_ = 500 °C was found to be ~35 times higher than that of their counterparts deposited at 25 °C, as shown in Figure 17b. Moreover, the observed dependence of the THz electric field amplitude on both incident laser power and azimuthal angle confirms the interplay between the transient photoexcited surge currents generated via the built-in surface depletion field and the photo-Dember effects, without excluding a possible contribution from electric field-induced optical rectification [52,55].

**Figure 18:**
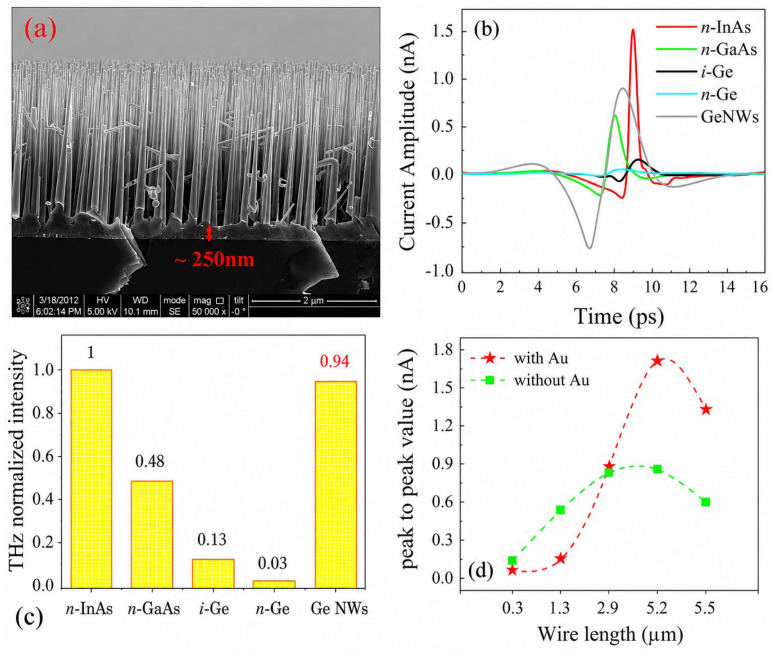
(**a**) Cross-sectional SEM image of Ge NWs after a growth time of 40 mins. (**b**) THz pulses emitted from various semiconductors. (**c**) A histogram comparing the peak-to-peak values of the THz pulses shown in (**b**,**d**). Peak-to-peak values of THz pulses of Ge NWs with/without Au as a function of their NW length. Reproduced from Ref. [44] under CC BY-NC-ND 3.0.

Apart from sputtering and other PVD techniques, chemical vapor deposition (CVD)-based approaches have also been used to grow various Ge nanostructures. Among these, low-pressure (LPCVD) [263] and plasma-enhanced (PECVD) [264] are frequently employed for Ge film growth. These CVD-based techniques generally rely on the decomposition of Ge-containing gaseous precursors, such as germane (GeH_4_), at elevated temperatures, typically between 600 °C and 800 °C. Of particular interest is the growth of Ge nanowires (NWs) in ultrahigh vacuum CVD systems via the vapor–liquid–solid (VLS) mechanism, where NWs are synthesized in a tapered shape with a steeple-like apex and a Au nanoparticle catalyst at the tip (e.g., Figure 18a) [44]. These n-type Ge NWs with varying lengths and wire densities have been reported to emit THz radiation upon their excitation with a Ti:Sapphire fs laser pulse, exhibiting a peak emission around ~0.2 THz for NWs with a length of 5.2 µm. The THz emission of the 5 µm-long Ge NWs was found to be twice that of n-GaAs and nearly comparable to that of n-InAs, as shown in the THz amplitude spectra in Figure 18b and the histogram peak-to-peak THz amplitudes in Figure 18c [44]. In addition, Figure 18d shows that the presence of Au nanoparticles at the tips of the Ge NWs enhances the THz emission, particularly for longer NWs (>2.9 µm), likely due to an increased built-in surface electric field. These results highlight that the dominant THz emission mechanisms in Ge NWs are strongly influenced by their geometrical properties, which provide an extended surface area for more efficient light absorption through multiple scattering pathways. This enhanced photon absorption leads to significant carrier separation at the NW surface, driven by either the built-in electric field or the photo-Dember effect. No contribution from optical rectification was reported in the case of Ge NWs [44]. Top-down nanostructuring of Ge surfaces has also been shown to be beneficial for THz emission.

**Figure 19:**
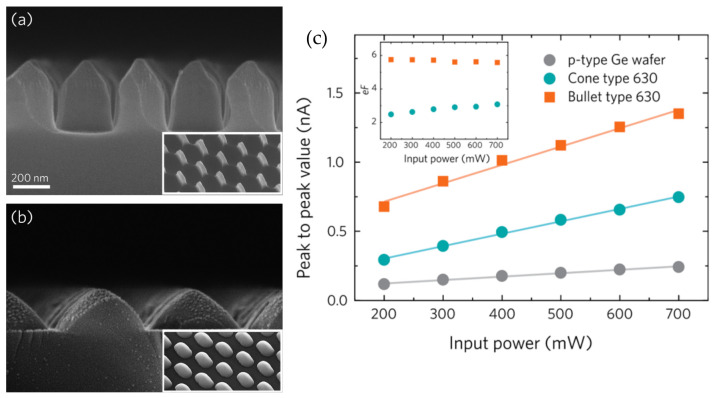
Cross-sectional SEM images of triangular arrays of (**a**) nano-bullets and (**b**) nano-cones with a period of 630 nm on p-type (100) Ge wafers; (**c**) incident laser power dependence of the THz signal emitted from nanobullets and nanocones shown in (**a**,**b**) along with that emitted by a flat b-type (100) Ge wafer. Reproduced from Ref. [58] with the permission of AIP Publishing.

Indeed, a significant enhancement of the THz emission from p-type (100) Ge wafers has been observed following plasma etching of their surface to form 2D hexagonal arrays of nano-bullets and nano-cones with a period of 630 nm using reactive ion etching (RIE) and inductively coupled plasma etching (ICP-RIE). The heights of the cones and the bullets were designed to be 227 nm and 399 nm, as shown in Figure 19a and Figure 19b, respectively [58]. While the peak-to-peak THz pulse amplitude was found to increase linearly with the input laser power, regardless of the surface nanostructuring, the nano-bullets clearly outperform the cones or the flat Ge wafer, as shown in Figure 19c [58]. This enhancement in THz radiation from Ge nanostructures is attributed to the increased effective charge separation at the surface. To further benchmark the THz radiation from these Ge nano-bullets, a comparison with an n-GaAs wafer under identical experimental conditions revealed comparable THz emission performance [58].

**Figure 20:**
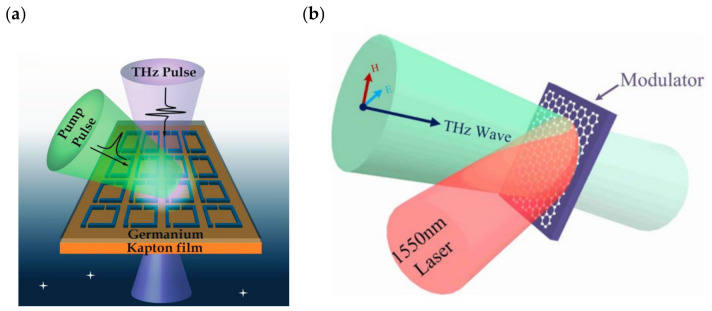
Schematic illustration of the (**a**) flexible Ge metaphotonic devices [265], and (**b**) the all-optical spatial THz modulator based on single-layer graphene on Ge (GOG) [266]. Reproduced from Ref. [265] with permission from © 2018 Wiley-Vch Verlag GmbH & Co. and [266] under CC BY-NC-SA 4.0.

Beyond surface nanostructuring, Ge thin film deposition on flexible Kapton substrates has also been reported for ultrafast optical switching of THz radiation. As shown in Figure 20a [265], a resonant transmission modulation depth of 90% is observed for THz asymmetric split-ring resonators (TASRs), with an ultrafast full recovery time of 17 ps and a decay constant of 670 fs, attributed to trap-assisted recombination in those thermally evaporated Ge films. In addition to the mechanical flexibility provided by the Kapton substrate, this device can also function as an active filter or an ultrafast modulator [265]. Furthermore, when a single layer of graphene is deposited on Ge (GOG), the resulting structure, driven by a 1.55 µm continuous-wave laser, can achieve a modulation depth of 94% with a modulation speed of ~200 kHz for THz transmission in the 0.25 to 1 THz range. As shown in Figure 20b, these GOG modulators are considered promising for integration with telecommunication fiber systems for broadband, high-speed, and low-cost spatial THz modulation [266].

Accessing a thorough comparison of the reported THz emission characteristics from different Ge-based emitter architectures, based on a standardized figure of merit, would have been ideal. However, the literature on THz emission from various Ge structures employs a wide range of experimental conditions, including differences in excitation wavelength, pulse duration, repetition rate, pump fluence, beam geometry, and THz detection techniques. All these variations limit direct quantitative comparison of absolute THz field amplitudes across independent studies, which are often reported in arbitrary or instrument-dependent units. Nevertheless, to partially address this limitation, Table 6 compiles representative results from different Ge fabrication approaches (including Czochralski growth, reactive ion etching, VLS-assisted CVD, and sputtering), emphasizing relative trends in THz emission performance under their respective experimental conditions (where sufficient experimental information is available, the incident pump fluence has been included or estimated from reported parameters to improve comparability). It is emphasized that the purpose of this compilation is not to provide an absolute ranking of THz performance across different studies, but rather to highlight relative trends (within the same experimental context) associated with different material architectures and fabrication methods. This approach enables a more structured comparison while acknowledging the inherent limitations arising from heterogeneous experimental methodologies in the literature.

The stability over time (and/or under high pump fluences) is another relevant criterion for Ge THz emitter technology assessment. Despite the growing number of studies reporting THz emission from bulk, nanostructured, and photoconductive Ge emitters, investigations of their long-term operational stability remain scarce. To date, most studies have focused on maximizing THz performance and attempting to elucidate emission mechanisms. For instance, carrier-lifetime engineering (via Au ion implantation into Ge PCAs) has been shown to be effective in suppressing carrier accumulation at high repetition rates and thereby improving operational stability of the Ge emitters [67]. On the other hand, sputtered Ge thin films investigated in our previous work [52] exhibited stable THz emission characteristics after storage periods exceeding 36 months, with no noticeable degradation in THz field amplitude performance. While this observation suggests good material stability, it does not constitute a systematic assessment of long-term operational reliability under continuous femtosecond-laser excitation. It is clear that at this stage, systematic studies addressing photostability, laser-induced aging, and long-term reliability under prolonged femtosecond-laser excitation are still lacking. As Ge-based THz technologies continue to mature, such investigations will be essential for assessing their practical deployment potential.

In sum, each fabrication technique discussed above presents trade-offs between film quality, process complexity, substrate compatibility, and integration feasibility. The choice of a given deposition method/fabrication process to grow Ge films/nanostructures fundamentally influences key material properties, including grain size, crystallographic orientation, carrier/defect density, surface morphology, and optoelectronic properties. All these characteristics, in turn, play a pivotal role in governing the optoelectronic response and transient photoexcited carrier dynamics essential for efficient THz radiation.

## 5. Emerging Applications of THz Radiation

**Figure 21:**
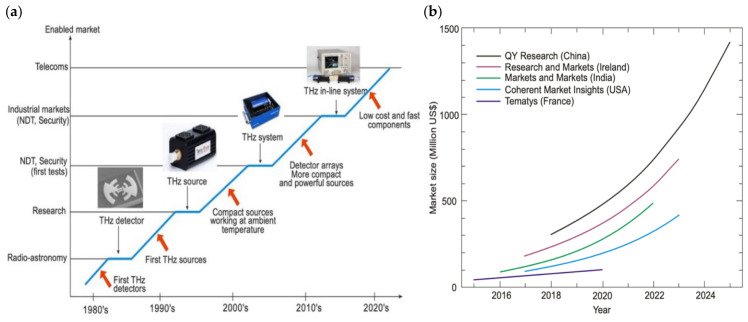
(**a**) Development of THz technologies and (**b**) global THz market evolution according to five different analyst firms. Reproduced from Ref. [267] under CC BY 4.0.

In the early 1980s, THz technology was primarily confined to laboratory use due to its high cost and demanding maintenance requirements. Since then, significant advances in optoelectronics have enabled the development of fully operational tabletop THz devices and components suitable for a wide range of applications, as illustrated in the timeline of Figure 21a [267]. Moreover, recent market surveys (Figure 21b) indicate growing commercial interest in THz technologies [267]. Photoconductive THz sources based on low- and wide-bandgap materials have significantly influenced the ‘lab-to-market’ ideology of efficient compact THz systems owing to their compactness, integrability, and ability to generate ultrafast pulses with tunable spectral properties. The intrinsic properties of these materials, such as their bandgap, carrier mobility, and recombination dynamics, play a crucial role in determining the performance of THz sources for practical applications. Furthermore, the relative transparency of many materials in the THz spectral range enables deep penetration into optically opaque or scattering media, thereby facilitating non-destructive testing and screening, imaging, and biological/chemical spectroscopy. These applications are extensively discussed in several comprehensive review articles dedicated exclusively to THz applications [41,75,268,269,270,271].

Beyond solid-state applications, THz radiation also plays an important role in gas-phase spectroscopy [272], Rydberg-atom physics [273], and astronomical observations [274], further illustrating the broad scientific relevance of this spectral region. All these applications collectively highlight the relevance and versatility of THz radiation for different socio-economic fields. With the emergence of CMOS-compatible materials such as Ge, GeSn, and layered 2D materials, the functional capabilities of THz sources are expected to expand further, bridging gaps across various sensing, communication, and emerging quantum technologies. The following three subsections highlight representative application domains that demonstrate the practical significance of semiconductor-based THz sources.

### 5.1. Non-Destructive Evaluation and Imaging

**Figure 22:**
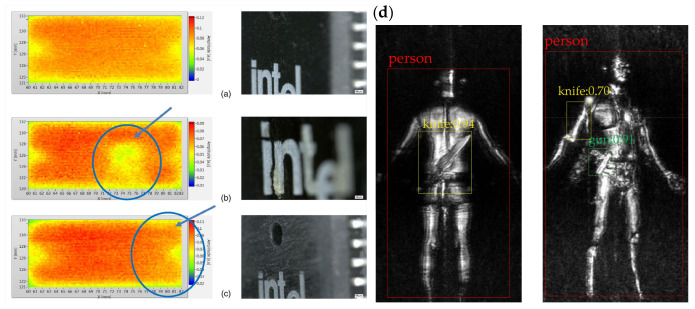
THz reflection images and their corresponding optical images of (**a**) an authentic IC. (**b**) A counterfeit recycled IC with the contaminated spot visible in the THz image. (**c**) IC that is sanded on one side [275]. (**d**) THz images revealing the identification of concealed suspicious objects (knife and gun) carried by a person [277]. Reproduced from Refs. [275,277] under CC BY 4.0.

Terahertz imaging has been widely used for numerous non-destructive evaluation and imaging applications, including aerospace inspection, security screening, and the identification of biological agents, chemical weapons, flammable substances, metallic and non-metallic weapons, and other potentially hazardous items. For instance, for on-chip inspection, THz reflection imaging can identify counterfeits, delamination, voids, and micro-cracks without disassembling the sample. The contrast between different materials, as shown in Figure 22a–c, arises primarily from variations in their THz absorption coefficients, ultimately enabling the distinction of internal features such as bond wires, die geometry, and different types of polymers or fillers [275]. Nonetheless, the image quality of THz imaging is generally lower than that of X-ray imaging; however, THz systems can operate at significantly higher acquisition speeds while presenting lower operational risks due to their non-ionizing nature and low photon energies. Additionally, THz transmission imaging can help identify suspicious objects, as shown in Figure 22d, enabling real-time, high-contrast detection of concealed metallic weapons, contraband (such as drugs and explosives), and other dielectric substances (e.g., water) beneath clothing [276,277].

### 5.2. Biochemical Imaging and Sensing

**Figure 23:**
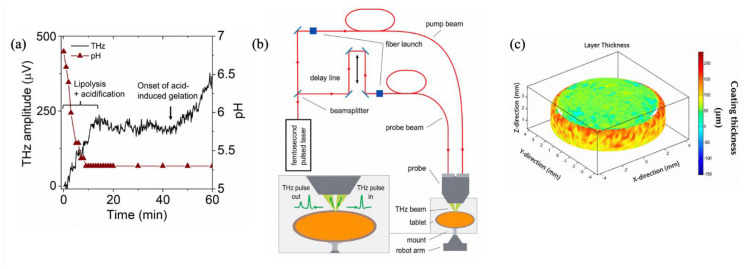
(**a**) Lipase activity in whole milk, where the changes in pH and THz amplitude were monitored using THz chemical microscopy (TCM) when exposed to the C. Antarctica lipase B (CALB) enzyme [286]. (**b**) Schematic of THz pulsed imaging (TPI). (**c**) 3D coating thickness image of one face and the center band of a biconvex tablet [288]. Reproduced from Ref. [286] under © 2021 Wiley & Sons, Inc., and from Ref. [288] under CC BY 4.0.

As mentioned above, THz radiation is particularly well suited for biomedical imaging owing to its low photon energy, non-ionizing nature, substantial penetration depth, and high sensitivity to water content and molecular resonances. Previous studies have shown that THz spectroscopy enables effective detection of blood glucose [278], cancerous and tumor tissues [279,280], dental caries [281], and skin anomalies [282] through the analysis of variations in THz absorption and refractive index. In addition, THz spectroscopy can elucidate amino acid signatures [283], DNA unwinding dynamics [284], and protein interactions [285], demonstrating strong potential for non-invasive medical diagnostics [286]. A novel technique, known as THz chemical microscopy (TCM), has recently been developed for the label-free characterization of important biomolecules such as proteins, DNA, and RNA [286,287]. Figure 23a shows the potential of the TCM in qualitatively monitoring the catalytic lipolysis process within complex samples such as whole milk when exposed to the C. Antarctica lipase B (CALB) enzyme [286]. Apart from sensing chemical changes within a complex sample, TCM has also exhibited sensitivity towards physical changes in the sample (acid-induced gelation after 40 min) that could be vital for certain industrial processes. On the other hand, THz pulsed imaging, schematically illustrated in Figure 23b, has been employed in the pharmaceutical industry to provide excellent contrast between coating structures, with μm-scale precision, despite the similar refractive indices of adjacent layers, as shown in Figure 23c [288].

Beyond the imaging of biomolecules, THz spectroscopy has also shown significant potential for chemical and gas sensing owing to its ability to probe low-frequency vibrational modes arising from inter- and intra-molecular motions, as well as rotational transitions, that are particularly valuable for the characterization of gaseous molecules. Polar compounds, including ammonia (NH_3_) [289], sulfur dioxide (SO_2_), hydrogen sulfide (H_2_S) [290], and formaldehyde (HCHO) [291], exhibit strong absorption lines in the THz region, enabling their selective detection at sub-ppm concentrations. These unique spectral fingerprints provide a non-invasive, label-free, and highly precise method for molecule identification using THz systems [292]. In addition to the detection of explosive compounds (such as AAZ, GUAZ, and TAGAZ) and caged materials (such as HNIW and TEX) [293], the THz-TDS systems have also exhibited sensitivity to a wide range of pollutants and greenhouse gases, thereby opening potential capabilities for security screening and environmental air-quality monitoring [292].

### 5.3. Art and Archeological Preservation

**Figure 24:**
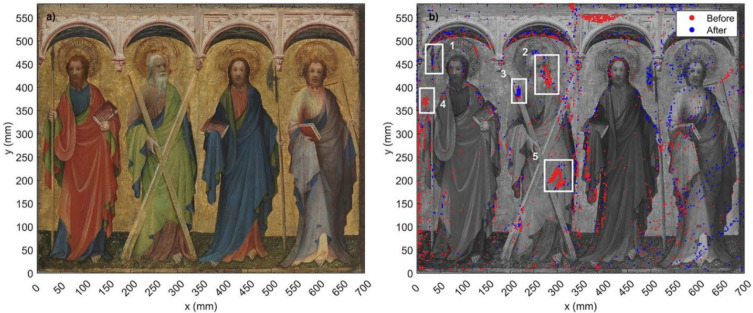
(**a**) Visible color photograph of The Apostles Philip, Andrew, Mattias and Thomas (c.1410-25). (**b**) THz mapping of delamination on the painting before (red) and after (blue) the consolidation effort. Delamination is denoted by dense groups of markers. Enclosed in white boxes (numbered 1 to 5) are the five regions of interest that were selected for further analysis. Reproduced from Ref. [294] under CC BY 4.0.

THz time-domain imaging (THz-TDI) is increasingly being employed in cultural heritage studies as a non-destructive and non-ionizing alternative to conventional imaging techniques. Unlike X-rays (with photon energies above 5–10 keV), which may induce radiation-related damage, THz radiation (with lower photon energies in the meV) is well suited for the examination of delicate materials such as pigments, inks, parchment, canvas, and other historical artifacts. Furthermore, the ability of THz waves to penetrate optically opaque dielectric layers enables depth-resolved visualization of subsurface structures and interfaces. For example, Figure 24 illustrates a representative example where an InGaAs-based THz-TDI system was used for the non-destructive identification and localization of delaminated regions in the artwork (highlighted by red and blue dots) [294]. Owing to its unique combination of non-ionizing operation, THz-TDI can be complemented by other conventional analytical techniques (such as cross-sectional imaging, Raman spectroscopy, Fourier transform infrared spectroscopy, and X-ray imaging) depending on the specific information sought, thereby enabling a more comprehensive diagnostic approach for art historians and conservation scientists [295,296].

## 6. Conclusions and Future Prospects

Despite the longstanding recognition of the technical foundations of THz radiation (dating back to the 1890s) [297], several intrinsic and extrinsic constraints continue to limit its widespread deployment in commercial and industrial applications. A major breakthrough occurred in the early 1990s with the development of far-infrared absorption and dispersion studies in crystalline dielectrics (e.g., sapphire, quartz, and silica) and semiconductors (e.g., Si, GaAs, and Ge) over the 0.2–2 THz range [298]. Since then, semiconductor-based THz sources have evolved into a multidisciplinary research domain rooted in ultrafast carrier dynamics and advanced microfabrication. Fundamental emission and detection mechanisms, including photoconductive switching and nonlinear transient charge-carrier acceleration, have established the physical framework of THz generation. Meanwhile, a wide range of materials, ranging from traditional III–V and II–VI semiconductors to emerging platforms (such as 2D layered materials, topological insulators, Weyl semimetals, hybrid perovskites, and group IV low-bandgap semiconductors), offer diverse opportunities due to their tunable optoelectronic properties. Nonetheless, THz emitters based on low- and wide-bandgap materials come with their own set of technological challenges. One of the primary concerns is the relatively low emitted THz signal intensity under practical operating conditions. This limitation often arises from reduced photocarrier generation efficiency, limited carrier mobility, rapid recombination dynamics, and saturation effects at high pump fluences. In ultrathin films, 2D materials, and nanostructures, the active volume available for photocarrier generation, acceleration, or nonlinear polarization is often inherently small, thereby restricting overall THz output. Additional degradation of THz emission performance may result from Joule heating, multiphoton absorption, and screening of internal fields under strong optical excitation conditions. Material-related imperfections further complicate device performance. Lattice defects, grain boundaries, strain-induced defects, and surface/interface states, particularly in polycrystalline or amorphous thin films, introduce trap states that significantly affect carrier lifetimes and recombination pathways. Such inhomogeneities hinder efficient charge-carrier acceleration, which is central to broadband far-field THz emission via transient photocurrent surge mechanisms. At the system level, the integration of THz sources with Si-based photonics and CMOS-compatible platforms remains a key challenge for scalable and cost-effective implementation. Although group IV semiconductors such as Ge and GeSn offer compatibility with Si-based processing, their growth still requires careful strain and defect management to preserve high crystallinity and optoelectronic performance. Similarly, hybrid approaches incorporating 2D materials [299] or plasmonic metamaterials [300] must address challenges related to process compatibility, thermal budgets, patterning scalability, and on-chip interconnect integration. Continued progress in monolithic integration strategies and photonic–electronic co-design will therefore be essential for the achievement of chip-scale THz systems.

Beyond device engineering, THz radiation provides a powerful probe of coherent charge dynamics, intervalley scattering, polaron formation, and spin–orbit coupling on fs timescales. In this context, semiconductor-based THz sources serve as a testbed for investigating intense nonlinear light–matter interactions beyond the perturbative regime, including nonlinear carrier acceleration and THz-induced phase transitions. These capabilities are expected to underpin the development of next-generation ultrafast switches, modulators, and compact CMOS-compatible THz systems. However, such applications require enhanced spectral tunability, precise control of THz waveforms, and integration with cryogenic and ultrafast optical systems, thereby imposing stringent demands on both material quality and device engineering.

From an application perspective, beyond ultrafast THz spectroscopy, emerging applications in biochemical imaging, chemical sensing, security screening, and art preservation continue to drive innovation at both the fundamental and technological levels. THz time-domain spectroscopy remains a key tool for probing rotational and vibrational modes, charge transport phenomena, and inter- and intra-valley scattering processes in materials. In the field of telecommunications, it is worth mentioning that THz technologies offer significant potential for meeting the ever-increasing demand for ultra-high-bandwidth wireless communications, particularly for point-to-point links operating over distances ranging from sub-meter scales to several tens of meters [301]. Their extremely high carrier frequencies provide access to spectral bandwidths far beyond conventional microwave and millimeter-wave regimes, enabling data rates potentially reaching the terabit-per-second range [302,303,304].

Overall, transient-photocurrent THz emission is governed by the combined influence of optical bandgap, photoexcited carrier mobility, carrier recombination lifetimes, intrinsic surface depletion fields, and device architecture. Group III–V materials remain the benchmark platforms for high-performance THz radiation, whereas group II–VI materials provide complementary nonlinear, electro-optic, and high-field capabilities. Emerging 2D, layered, topological insulators, and Weyl semimetals further expand the existing THz technological landscape via electrical tunability and unconventional photocurrent mechanisms. Nevertheless, their practical implementation remains constrained by material quality, limited active volume, reproducibility, and scalability. However, group IV THz emitters remain comparatively underexplored in the existing literature despite their clear advantages for CMOS-compatibility and wafer-scale manufacturability. This gap motivated the particular emphasis placed in this review article on Ge-based THz emitters, whose tunable bandgap, high carrier mobilities (as compared to Si), and technological maturity position Ge as a particularly promising platform for future compact, CMOS-compatible, and efficient THz sources. Overall, continued progress in semiconductor materials engineering (including optimized and scalable synthesis, defect and strain control, and heterogeneous integration of different semiconductors) is going to drive the progress of next-generation THz devices from laboratory prototypes to deployable technologies.

## Figures and Tables

**Table 2:** Comparison of group II–VI materials used for THz radiation, including their band gaps, carrier mobilities (at room temperature), dominant THz mechanisms, and key advantages and limitations. The reported values are representative and may vary with composition, crystal quality, nanostructuring, growth conditions, defect density, and device architecture.

Material	Bandgap (eV)	Electron/Hole Mobility (cm^2^ V^−1^ s^−1^)	Dominant THz Mechanism(s)	Key Advantages	Key Limitations	Refs.
**ZnTe**	~2.2	~340/~100	Optical rectification	Excellent EO coefficient, standard THz EO crystal	Phase-matching, phonon absorption	[30,137,150]
**CdTe**	~1.5	~1100/~100	Surface-field emission, photo-Dember effect	Strong nonlinear response, plasmon-enhanced THz	Strong pump absorption, phase mismatch near band-edge	[138,139,151,152]
**HgTe NCs**	~0.3–1	Size-dependent	Photogalvanic and photon drag effect	Tunable THz response, quantum-confinement	Surface-trap assisted recombination	[142,143]
**Hg_1−x_Cd_x_Te (MCT)**	~0.2–1.6	~20,000 (for Hg-rich)	Photoconductive response, hot-electron effects	Tunable bandgap, highly sensitive	Complex growth, requires cryogenic operation	[145,153,154]
**ZnSe**	~2.6	~530/~30–50	Biased photoconductive emission	High dielectric breakdown strength, suitable for LAPCAs	Lower carrier mobility than GaAs, thermal saturation	[94,155,156]

**Table 5:** A non-exhaustive comparison list of some topological insulators (TIs) and Weyl semimetals (WSMs) used for THz generation, including their band gaps, carrier mobilities, dominant THz-emission mechanisms, and key advantages and limitations. The reported values are representative and may vary with composition, crystalline orientation, defect density, and chemical ordering.

Material	Bandgap (eV)	Electron/Hole Mobility (cm^2^ V^−1^ s^−1^)	Dominant THz Mechanism(s)	Key Advantages	Key Limitations	Refs.
**Bi_2_Se_3_**	~0.2–0.3	~10^2^–10^3^/ ~275–500	Surface-depletion drift current, optical rectification	Robust topological surface states, broadband THz emission	Strong doping sensitivity, surface oxidation	[207,208,216,217]
**Bi_2_Te_3_**	~0.13–0.17	~1100–1250/ ~600–700	Photogalvanic effect, nonlinear injection surface currents	Strong spin–orbit coupling, chiral THz response	Sensitive to stoichiometry, oxidation and interface quality	[211,212,213,218]
**Bi_1−x_Sb_x_**	Composition dependent	~10^4^/~10^3^	Surface-field depletion, photo-Dember, OR	Tunable topological phase, strong spin-charge conversion	Strong dependence on crystallinity and orientation	[107,219,220]
**Sb_2_Te_3_**	~0.2–0.3	~10^2^–10^3^/ ~100–340	Linear/circular photogalvanic surface photocurrents	Helicity and polarization control, spin-momentum-locked response	Strong dependence on thickness and morphology	[214,215,221,222]
**TaAs**	Gapless semimetal	-	Circular photogalvanic effect, linear photogalvanic currents	Strong broadband and chiral response	Crystal growth complexity, fabrication scalability	[64,223,224,225]
**Co_2_MnGa**			Anomalous-Hall effect (AHE) induced transverse current	Efficient ultrafast spin-current generation	Strong dependence on chemical ordering, interface asymmetry	[226,227]
**Co_2_MnAl**			Inverse spin-Hall transverse current	Magnetically tunable AHE, short electron-spin relaxation times	Strong dependence on chemical ordering, crystalline phase	[227,228]

**Table 6:** Comparison of the relative THz emission enhancement from various Ge architectures fabricated via different fabrication methods and measured under varying excitation and detection conditions.

Architecture	Excitation Conditions [Wavelength (λ), Pulse Duration (fs)]	Laser Fluence (μJ/cm^2^)	Relative Enhancement	Key Limitations	Refs.
**p-Ge/n-Ge (111) wafers**	820 nm, 150 fs	-	n-Ge > p-Ge (by ∼3× times)	Limited wafer tunability	[54]
**Ge nano-bullets using RIE**	800 nm, 190 fs	~0.13	∼5× higher than bulk wafer	Ion-induced damage/Sidewall bowing	[58]
**Ge nano-cones using ICP-RIE**	800 nm, 190 fs	~0.13	∼3× higher than bulk wafer	Ion-induced damage/Surface roughness	[58]
**Sputtered a-Ge films**	800 nm, 50 fs	~0.75	Up to ∼4× enhancement	Poor crystallinity/High resistivity	[111]
**VLS grown Ge NWs**	790 nm, 190 fs	~0.04	Up to ∼1.8× enhancement	Difficult large-area uniformity and integration	[44]
**Sputtered poly-Ge films**	1035 nm, 350 fs	~3.80	∼26× higher than a-Ge films	Sensitive to crystallinity and deposition conditions	[52]

## Data Availability

No new data were created or analyzed in this study. Data sharing is not applicable to this article.

## Abbreviations

The following abbreviations are used in this manuscript:

**Abbreviation**	**Full form**
2D	Two-dimensional
3PA	Three-photon absorption
AAZ	3-Azido-1,2,4-triazole
AHE	Anomalous Hall Effect
BNA	N-benzyl-2-methyl-4-nitroaniline
BP	Black Phosphorus
BWO	Backward Wave Oscillator
CALB	Candida antarctica lipase B
CMOS	Complementary Metal–Oxide–Semiconductor
CW	Continuous Wave
CVD	Chemical Vapor Deposition
DAST	4-N,N-dimethylamino-4′-N′-methyl-stilbazolium tosylate
DFG	Difference Frequency Generation
DNA	Deoxyribonucleic Acid
DS	Dirac Semimetal
DSTMS	4-N,N-dimethylamino-4′-N′-methyl-stilbazolium 2,4,6-trimethylbenzenesulfonate
EFIOR	Electric Field-Induced Optical Rectification
EM	Electromagnetic
EO	Electro-Optic
FEL	Free-Electron Laser
FET	Field-Effect Transistor
fs	Femtosecond
Ge	Germanium
GeSn	Germanium–Tin
GOG	Graphene-On-Germanium
GUAZ	Guanidinium Azide
HCHO	Formaldehyde
HMQ-TMS	2-(4-Hydroxystyryl)-3-methylbenzothiazolium 2,4,6-trimethylbenzenesulfonate
HNIW	Hexanitrohexaazaisowurtzitane
ICP-RIE	Inductively Coupled Plasma Reactive Ion Etching
IMPATT	Impact Ionization Avalanche Transit-Time
IR	Infrared
LAPCA	Large-Aperture Photoconductive Antenna
LH	Light Hole
LPCVD	Low-Pressure Chemical Vapor Deposition
LSPR	Localized Surface Plasmon Resonance
LT-GaAs	Low-Temperature-Grown Gallium Arsenide
MBE	Molecular Beam Epitaxy
MCT	Mercury Cadmium Telluride
MoS_2_	Molybdenum Disulfide
MoSe_2_	Molybdenum Diselenide
NC	Nanocrystal
NDR	Negative Differential Resistance
NIR	Near-Infrared
NPG	Nanoporous Gold
NW	Nanowire
OH1	2-(3-(4-Hydroxystyryl)-5,5-dimethylcyclohex-2-enylidene)malononitrile
OPO	Optical Parametric Oscillator
OR	Optical Rectification
PCA	Photoconductive Antenna
PECVD	Plasma-Enhanced Chemical Vapor Deposition
PD	Photo-Dember
PLD	Pulsed Laser Deposition
PPV	Poly(p-phenylene vinylene)
ps	Picosecond
QCLs	Quantum Cascade Laser
RIE	Reactive Ion Etching
RNA	Ribonucleic Acid
RT	Room Temperature
RTD	Resonant Tunneling Diode
SF_6_	Sulfur Hexafluoride
SHG	Second Harmonic Generation
Si	Silicon
SNR	Signal-to-Noise Ratio
SO_2_	Sulfur Dioxide
TASR	Terahertz Asymmetric Split-Ring Resonator
TCM	Terahertz Chemical Microscopy
TES	Terahertz Emission Spectroscopy
THz	Terahertz
THz-TDI	Terahertz Time-Domain Imaging
THz-TDS	Terahertz Time-Domain Spectroscopy
TIs	Topological Insulators
TMDCs	Transition Metal Dichalcogenides
TRL	Technology Readiness Level
VDW	van der Waals
VLS	Vapor–Liquid–Solid
WSMs	Weyl Semimetals
ZnSe	Zinc Selenide
ZnTe	Zinc Telluride
